# Association of long-term exposure to various ambient air pollutants, lifestyle, and genetic predisposition with incident cognitive impairment and dementia

**DOI:** 10.1186/s12889-024-17702-y

**Published:** 2024-01-15

**Authors:** Rongguang Ge, Yue Wang, Zengli Zhang, Hongpeng Sun, Jie Chang

**Affiliations:** 1 School of Public Health, Suzhou Medical College, Soochow University, 199 Renai Road, Suzhou, Jiangsu 215123 China; 2 Department of Neurology, Second Affiliated Hospital of Soochow University, Suzhou, Jiangsu 215004 China

**Keywords:** Cognitive impairment, Dementia, Air Pollution, Lifestyle, Gene, UK Biobank

## Abstract

**Background:**

Long-term exposure to air pollution has been found to contribute to the development of cognitive decline. Our study aimed to assess the association between various air pollutants and cognitive impairment and dementia. Additionally, explore the modification effects of lifestyle and genetic predisposition.

**Methods:**

The exposure levels to various air pollutants, including particulate matter (PM) with diameters ≤ 2.5 (PM_2.5_), ≤ 10 (PM_10_), and between 2.5 and 10 μm (PM_2.5−10_) and nitrogen oxides (NO and NO_2_) were identified. An air pollution score (APS) was calculated to evaluate the combined exposure to these five air pollutants. A genetic risk estimate and healthy lifestyle score (HLS) were also generated. The Cox regression model adjusted by potential confounders was adopted to access the association between pollution exposure and cognitive decline, and several sensitivity analyses were additionally conducted to test the robustness.

**Results:**

The combined exposure to air pollutants was associated with an increased risk of incident cognitive decline. Compared with the low exposure group, the hazard ratio (HR) and 95% confidence interval (CI) for all-cause dementia, Alzheimer’s dementia, vascular dementia, and mild cognitive impairment (MCI) in those exposed to the highest levels of air pollutants were respectively 1.07 (95% CI: 1.04 to 1.09), 1.08 (95% CI: 1.04 to 1.12), 1.07 (95% CI: 1.02 to 1.13), and 1.19 (95% CI: 1.12 to 1.27). However, the modification effects from genetic predisposition were not widely observed, while on the contrary for the healthy lifestyle. Our findings were proven to be reliable and robust based on the results of sensitivity analyses.

**Conclusions:**

Exposure to air pollution was found to be a significant contributing factor to cognitive impairment and dementia, and this association was not easily modified by an individual’s genetic predisposition. However, adopting a healthy lifestyle may help to manage the risk of cognitive decline related to air pollution.

**Supplementary Information:**

The online version contains supplementary material available at 10.1186/s12889-024-17702-y.

## Introduction

Ambient air pollution, as a complex mixture of particulate matter (PM), gases, organic compounds, and metals [1], has become the largest environmental cause of disease and premature death in the world today [2]. The data from the Lancet Commission showed that air pollution was responsible for approximately 9 million originally avoidable cases of premature death in 2015 alone, causing three times more deaths than that from acquired immune deficiency syndrome (AIDS), tuberculosis, and malaria combined [2], suggesting that air pollution is a current and ongoing threat to the public health around the world.

Cognitive impairment generally refers to a certain degree of cognitive dysfunction caused by various factors, ranging from mild cognitive impairment (MCI) to severe dementia. Nowadays, cognitive impairment has become a worldwide health issue, leading to grave disability for patients and placing a heavy burden on their family caregivers. According to the estimation [3], there were about 35.6 million people suffering from dementia around the world in 2010, with its number almost doubling every 20 years, which means nearly 115.4 million people might have to live with dementia by 2050.

In recent years, growing evidence from publications [4, 5] concerning the association between air pollution and brain function indicated the ability of air pollutants to cast adverse effects on the central nervous system, causing various neurodegenerative diseases. Additionally, both lifestyle [6] and genetic predisposition [7] were reported to be related to the incidence of cognitive impairment. Yet unfortunately, although former researchers have confirmed air pollution exposure, lifestyle, and genetic predisposition could independently influence an individual’s cognitive level, whether lifestyle and genetic predisposition may modify the air pollution-induced cognitive impairment remained unclear. Therefore, in pursuit of filling this academic gap, our research team aimed to fully analyse and describe the association of both combined and single exposure to various air pollutants with cognitive impairment and dementia, and further explore the potential modification effects caused by lifestyle and genetic predisposition.

This current study was completely based on the comprehensive data on ambient air pollutants, lifestyle, and genetic variety provided by the UK Biobank. Using an air pollution score (APS), which was the result of the joint assessment of five different pollutants, including PM with diameters ≤ 2.5 (PM_2.5_), ≤ 10 (PM_10_), and between 2.5 and 10 μm (PM_2.5−10_) and nitrogen oxides (NO and NO_2_), researchers were able to perform a systematic analysis in quintiles. Meanwhile, the population attributable fraction (PAF) was also calculated to explicate the proportion of cognitive decline patients that could be attributed to air pollution, making our study more accurate and rigorous.

## Methods

### Study population

UK Biobank, as a large population-based, nationwide, and open-access prospective study, recruited over 500,000 individuals in conjunction with 22 assessment centres across the UK. Through self-completed touch-screen questionnaires, computer-assisted interviews, physical and functional measurements, and samples of blood, urine, and saliva, it successfully collected a large variety of health-related information, consisting of sociodemographic characteristics, diseases phenotypic, lifestyle, and genetic variants [8, 9]. More details of the study design and methodology have been thoroughly described by former researchers [8]. UK Biobank is operating under the approval of North-West Multicentre Research Ethics Committee to ensure its ethical robustness. All participants provided their consent for regular blood, urine, and saliva sampling and more accurate data on their lifestyles. Furthermore, the dataset also promised to be anonymized to protect the privacy of participants.

In the present study, the inclusion and exclusion criteria were set as follows. Inclusion: (1) participants who gave written consent to participate; (2) participants who have completed the follow-up. Exclusion: (1) participants who had a history of cognitive impairment or dementia on the basis of self-report or medical records at the baseline visit; (2) participants with missing air pollution exposure information; (3) participants with missing genetic data; (4) participants with missing lifestyle information. For the duration of follow-up, according to the UK Biobank, the recruitment of participants was carried out between 2006 and 2010, while the end date of follow-up was December 31, 2019. As a result, we excluded those with MCI or any form of dementia at the baseline. After the screening, a total of 502,149 participants were included for consideration. However, 41,277 of them were found to lack the necessary information about air pollution exposure and were eventually excluded, leaving a total of 460,872 eligible participants for the final analysis. The flow chart of study participants was displayed in Supplementary Fig. S1.

### Assessment of outcomes

All patients were diagnosed in accordance with the criteria of the International Classification of Diseases, 10th revision (ICD-10). The ICD-10 codes of all-cause dementia included G30, F01, G20. The ICD-10 codes of Alzheimer’s dementia, vascular dementia, and MCI were set as G30, F01, and F06.7 respectively. More detailed information was shown in Supplementary Table S1.

### Air pollution score (APS)

With its ability to consider different types of land-use variables in the assessment of target pollutants, land-use regression has now become an effective means to depict intra-urban air pollution concentration variation in fine spatial-temporal resolution globally [10]. Therefore, the UK Biobank Study adopted a land-use regression model based on the European Study of Cohorts for Air Pollution Effects (ESCAPE) project [11, 12] to estimate the annual average concentrations of PM_2.5_, PM_10_, PM_2.5−10_, NO_2_, and NO, which can be found at https://biobank.ndph.ox.ac.uk/showcase/label.cgi?id=114. While the land-use regression models calculate the annual moving average concentrations of air pollutants using the predictor variables retrieved from the GIS variables, including land use, traffic, and topography by a 100 m × 100 m resolution. Participants’ ambient air pollution concentrations were then assigned according to their residential coordinates in the 100 m × 100 m grid cells. The exposure levels of five air pollutants that mentioned above were all collected in 2010.

In order to further assess the combined exposure of five different ambient air pollutants, we calculated an APS [13] by summarizing the concentrations of PM_2.5_, PM_10_, PM_2.5−10_, NO_2_, and NO, weighted by the multivariable-adjusted risk estimates (β coefficients) on cognitive impairment in the current study. The equation was:

$$\begin{array}{l}{\text{Air pollution score}}\\ = ({\beta _{PM2.5}} \times P{M_{2.5}} + {\beta _{PM10}} \times P{M_{10}} + {\beta _{PM2.5 - 10}} \times P{M_{2.5 - 10}} + {\beta _{N{O_2}}} \times N{O_2}\\ \quad + {\beta _{NO}} \times NO) \times (5 \div {\text{sum of the }}\beta \,{\text{coefficients}})\end{array}$$

The APS ranged from 39.22 to 157.77. A greater score indicated a higher level of combined exposure to various air pollutants. All participants were categorized as five groups according to the quintiles of the APS level.

### Evaluation of genetic risk

The genetic association of dementia has been widely confirmed by evidence from mounting publications based on genome-wide association study (GWAS) [14–19]. In the UK Biobank project, research team conducted the genotyping, imputation, and quality control of the genetic data, providing a critical route to further investigation into the heredity-related dementia. More specific descriptions were available elsewhere [20].

According to the previous studies [21, 22], the expression of polymorphisms in the apolipoprotein E gene (APOE) was recognized as a strong genetic risk factor for Alzheimer’s dementia and several other neurodegenerative diseases, including vascular dementia and Lewy body dementia. Therefore, in the present study, the APOE status recorded in the genetic database of UK Biobank were fully utilized by researchers to evaluate the genetic risk for cognitive impairment among participants. The population was divided into three groups of low, intermediate, and high genetic risk of cognitive impairment based on their APOE gene carrying status for the convenience of subsequent statistical analysis.

### Healthy lifestyle score (HLS)

A healthy lifestyle score (HLS) was generated based on 7 variables: physical activity, body mass index (BMI), alcohol consumption, smoke status, waist-to-hip ratio (WHR), sedentary time (hours/day), and vegetable and fruit intake (servings/day).

The BMI was calculated as weight divided by height squared (kg/m^2^). On the basis of multiple meta-analyses [23–27] on BMI-related all-cause mortality, healthy weight was defined as the BMI values in a normal range (18.5 ~ 24.9); As regards the physical activity estimation of an individual, the International Physical Activity Questionnaire (IPAQ) guideline [28, 29] was adopted for metabolic equivalent task (MET) calculation. A physical activity guideline [30] was also used to determine whether the MET values were appropriate for the benefits of participants’ health; The WHR was defined as waist circumference (WC) divided by hip circumference (HC), which was a strong indicator of central obesity. According to the recommendation from IPAQ guideline [28], the WHR was identified as healthy when it was < 0.85 for females, and < 0.90 for males; The time spent using the computer and watching television for recreational purposes was calculated as sedentary time. Participants were classified as unhealthy if they failed to keep the daily sedentary time less than 3 h [31]; The participants reported their dietary arrangements at the baseline visit for the calculation of the total fruits and vegetables intake, with ≥ 6 servings/day categorized as healthy diet [32]; In terms of alcohol and tobacco consumption, the population was divided into three groups of never, previous, and current, among which that currently smoking or drinking was identified as unhealthy living habit.

Participants scored 1 point for each of these health-related behaviours once they met the criteria mentioned above. The HLS ranged from 0 to 7 theoretically. After the scoring was completed, participants were split up into three groups as unfavourable (0, 1), intermediate (2, 3), and favourable (≥ 4) in accordance with their HLSs.

### Measurements of other potential covariates

Research team collected age, sex, ethnicity, Townsend deprivation index (TDI) [33], blood pressure level, employment status, education background, income bracket, and history of hypertension, diabetes, cardiovascular disease (CVD), coronary artery disease (CAD), and stroke as potential modification factors.

During the initial baseline visit, both systolic blood pressure (SBP) and diastolic blood pressure (DBP) were measured by trained clinical workers to ascertain the blood pressure level. In addition, the income bracket evaluation was based on the average total household income (<£18,000, £18,000~£52,000, £52,000~£100,000, and >£100,000). The data on education duration was used for the assessment of education background (≤ 7 years, 8 ~ 10 years, 11 ~ 15 years, and ≥ 16 years).

### Statistical analysis

The follow-up time was measured from the recruitment date to the first diagnosis of any form of cognitive impairment or dementia, lost to follow-up, death, or end of the current follow-up, whichever came first. Our research team adopted Cox proportional hazards models to evaluate the hazard ratio (HR) and 95% confidence interval (CI) for the incident cognitive impairment and dementia related to single air pollutants and the APS. Other potential confounders, including age (continuous), sex (male, female), ethnicity (white, non-white), SBP (continuous), BMI (kg/m^2^, continuous), employment status (yes, no), physical activity (MET, continuous), education background (≤ 7 years, 8 ~ 10 years, 11 ~ 15 years, and ≥ 16 years), income bracket (<£18,000, £18,000~£52,000, £52,000~£100,000, and >£100,000), alcohol consumption status (never, previous, and current), tobacco consumption status (never, previous, and current), hypertension history (yes, no), diabetes history (yes, no), and CVD history (yes, no) were adjusted in those models that mentioned above.

Several sensitivity analyses were also performed to test the robustness of our outcomes. First, in order to reduce the impact of the missed diagnosis at the baseline visit on the effectiveness of analysis as much as possible, researchers excluded those cases which reported the cognitive impairment diagnosis in the first 2 years of follow-up. Second, an analysis of participants who have lived in the current address for at least 5 years was also conducted by the research team to estimate the long-term effect of air pollution exposure on cognitive impairment. Finally, researchers brought other mixed covariates, for example, age, sex, ethnicity, and so on, into consideration to minimize their potential influences during the follow-up.

Besides, given that not all participants diagnosed with cognitive impairment or dementia were necessarily attributed to air pollution exposure, our research team adopted Levin’s formula [34] to estimate the proportion of patients that could be prevented if the risk factor was eliminated. In the current study, the HRs were used as the risk ratios (RRs) for the calculation [35].

$$ Population\ Attributable\ Fraction \left(PAF\right)=\frac{{P}_{e}\times \left({RR}_{e}-1\right)}{\left[{P}_{e}\times \left({RR}_{e}-1\right)+1\right]}$$

Where P_e_ was the representation of risk factor prevalence and RR_e_ was the relative risk because of the factor, comparing the incidence of cognitive decline in the exposed and unexposed groups. However, participants may not only face a single risk factor. As a result, it was important to calculate the weighted PAF adjusted for the correlation to further account for the existence of multiple risk factors [36]. The formula [37] was shown as follows, where communality was the sum of the square of all factor loadings and the w was 1 minus communality.

$$ w=1-communality$$

$$ Weighted\ PAF=1-\prod \left[1-\left(w\times PAF\right)\right]$$

Meanwhile, the restricted cubic spline analysis was also used to examine whether there was a dose-response relationship between single air pollutants or the APS with the incidence of cognitive impairment and dementia. We additionally conducted the stratified analysis on potential confounders to further explore the possible relevance of the genetic predisposition, sociodemographic characteristics, lifestyle, and prevalent disease with air pollution-induced cognitive impairment.

All statistical analyses were performed with SAS software. All P-values were based on the two-sided test, and P-values < 0.05 were considered statistically significant. The figures in the current article were generated with R software, GraphPad Prism software (version 9.4.1), and Adobe Illustrator software.

## Results

### Descriptive analysis

Among 460,872 cohort participants without cognitive impairment and dementia at baseline and completed the measurements of potential covariates during the follow-up, a total of 7,840 patients diagnosed as cognitive impairment or dementia were identified at the end of the study, where 6,996 (89.23%) patients were attributed to all-cause dementia, 2,927 (37.33%) patients were attributed to Alzheimer’s dementia, 1,544 (19.69%) patients were attributed to vascular dementia, and 844 (10.77%) patients were attributed to MCI. The baseline characteristics of the all-cause dementia group in accordance with the quintiles of the APS were available in Table 1. The baseline characteristics of other three cognitive impairment and dementia subtypes were shown in Supplementary Table S2, S3, and S4 respectively. The study population was subsequently divided into five groups according to their APSs, ranging from 39.22 to 49.82, 49.82 to 54.61, 54.61 to 58.38, 58.38 to 63.09, and 63.09 to 157.77 respectively. As the score rose, participants tended to be younger and had greater TDI, indicating they were facing a higher level of deprivation and poverty [33]. Meanwhile, compared with low exposure group, participants in high exposure group were more likely to be current smokers, with a greater possibility of prevalent disease history, and less likely to perform physical activity, leading to a slight increase in BMI level.

**Table 1:** Baseline characteristics of the UK Biobank participants (*N* = 460,872) with all-cause dementia in accordance with the quintiles of air pollution score

	Air Pollution Score				
	Q1 (39.22, 49.82)	Q2 (49.82, 54.61)	Q3 (49.82, 54.61)	Q4 (58.38, 63.09)	Q5 (63.09, 157.77)
Follow-up duration, years	12.5 (1.7)	12.5 (1.8)	12.4 (1.8)	12.4 (1.9)	12.4 (2)
Age, years	57.8 (7.8)	57.6 (8)	57.3 (8.1)	56.7 (8.2)	56 (8.3)
Sex, n (%)					
Male	41,958 (45.5)	41,927 (45.5)	41,992 (45.6)	41,999 (45.6)	42,611 (46.2)
Female	50,217 (54.5)	50,247 (54.5)	50,183 (54.4)	50,175 (54.4)	49,563 (53.8)
Ethnicity, n (%)					
White	90,489 (98.2)	89,140 (96.7)	87,659 (95.1)	84,528 (91.7)	80,639 (87.5)
Non-white	1,686 (1.8)	3,034 (3.3)	4,516 (4.9)	7,646 (8.3)	11,535 (12.5)
TDI	-2.9 (2)	-2.4 (2.5)	-1.8 (2.7)	-0.8 (2.8)	1.2 (3.3)
BMI, kg/m^2^	27.1 (4.5)	27.4 (4.7)	27.5 (4.8)	27.6 (4.9)	27.6 (5.1)
MET	2711.2 (2725.6)	2643.5 (2681.8)	2664.6 (2723.2)	2679.8 (2757.2)	2639.9 (2724)
Waist-to-hip ratio	0.9 (0.1)	0.9 (0.1)	0.9 (0.1)	0.9 (0.1)	0.9 (0.1)
SBP, mmHg	140.3 (21.3)	139.8 (21.2)	139.5 (21.3)	139.3 (21.9)	138.3 (22.5)
DBP, mmHg	83.6(13)	83.4 (13)	83.4(13.1)	83.6(13.7)	83.6(14.5)
NO, µg/m^3^	27 (4.6)	36.4 (4.1)	42.3 (4.1)	48.6 (4.7)	65.8 (17.3)
NO_2_, µg/m^3^	17.2 (2.7)	22.7 (2.3)	26.3 (2.4)	29.9 (2.5)	37.2 (6.3)
PM_2.5_, µg/m^3^	8.7 (0.4)	9.5 (0.3)	9.9 (0.3)	10.4 (0.4)	11.5 (0.9)
PM_10_, µg/m^3^	14.3 (1.6)	15.8 (1.3)	16.4 (1.3)	16.9 (1.5)	17.8 (1.8)
PM_2.5−10_, µg/m^3^	6.2 (0.8)	6.2 (0.8)	6.3 (0.8)	6.5 (0.9)	6.9 (1)
Air Pollution Score	45.4 (3.1)	52.4 (1.4)	56.5 (1.1)	60.5 (1.3)	69.7 (7.1)
Employment, n (%)					
Yes	51,544 (55.9)	51,653 (56)	52,611 (57.1)	54,419 (59)	55,579 (60.3)
No	40,631 (44.1)	40,521 (44)	39,564 (42.9)	37,755 (41)	36,595 (39.7)
Education, n (%)					
≤7 years	12,286 (13.3)	15,254 (16.5)	16,975 (18.4)	17,504 (19)	17,728 (19.2)
8 ~ 10 years	15,380 (16.7)	16,755 (18.2)	16,897 (18.3)	16,333 (17.7)	14,575 (15.8)
11 ~ 15 years	18,122 (19.7)	17,222 (18.7)	16,250 (17.6)	15,391 (16.7)	14,285 (15.5)
≥16 years	46,387 (50.3)	42,943 (46.6)	42,053 (45.6)	42,946 (46.6)	45,586 (49.5)
Income, n (%)					
<£18,000	16,289 (17.7)	20,688 (22.4)	23,730 (25.7)	25,607 (27.8)	29,222 (31.7)
£18,000~£52,000	48,135 (52.2)	48,790 (52.9)	48,031 (52.1)	46,472 (50.4)	42,733 (46.4)
£52,000~£100,000	21,389 (23.2)	18,359 (19.9)	16,859 (18.3)	16,296 (17.7)	15,040 (16.3)
>£100,000	6,362 (6.9)	4,337 (4.7)	3,555 (3.9)	3,799 (4.1)	5,179 (5.6)
Smoke, n (%)					
Never	53,418 (58)	52,007 (56.4)	50,760 (55.1)	49,659 (53.9)	46,580 (50.5)
Previous	31,956 (34.7)	32,267 (35)	32,088 (34.8)	31,825 (34.5)	32,245 (35)
Current	6,801 (7.4)	7,900 (8.6)	9,327 (10.1)	10,690 (11.6)	13,349 (14.5)
Drink, n (%)					
Never	2,737 (3)	3,510 (3.8)	4,215 (4.6)	4,957 (5.4)	6,048 (6.6)
Previous	2,524 (2.7)	2,878 (3.1)	3,278 (3.6)	3,548 (3.8)	4,285 (4.6)
Current	86,914 (94.3)	85,786 (93.1)	84,682 (91.9)	83,669 (90.8)	81,841 (88.8)
Hypertension, n (%)	23,728 (25.7)	25,210 (27.4)	25,650 (27.8)	25,800 (28)	25,727 (27.9)
Diabetes, n (%)	3,697 (4)	4,483 (4.9)	4,785 (5.2)	5,194 (5.6)	5,659 (6.1)
CVD, n (%)	12,740 (13.8)	13,850 (15)	14,274 (15.5)	14,477 (15.7)	14,642 (15.9)
CAD, n (%)	10,375 (11.3)	11,476 (12.5)	11,822 (12.8)	12,025 (13)	12,026 (13)
Stroke, n (%)	3,281 (3.6)	3,517 (3.8)	3,636 (3.9)	3,658 (4)	3,932 (4.3)

Based on the Pearson correlation analysis, all single air pollutants (PM_2.5_, PM_10_, PM_2.5−10_, NO_2_, and NO) were found to be positively correlated with each other (Pearson correlation coefficients ranged from 0.20205 to 0.92211, all P-values < 0.0001). The highest correlation was detected between NO_2_ and NO (Pearson correlation coefficient = 0.92211, P-value < 0.0001). More detailed information was shown in Supplementary Table S5.

### Association between air pollution and cognitive decline

In terms of the association between single air pollutants and cognitive impairment, the impact of single PM_2.5−10_ exposure on cognitive decline was inconspicuous. While on the contrary, NO, NO_2_, PM_2.5_, and PM_10_ exposure were considered as significantly correlated with all-cause dementia, Alzheimer’s dementia, and MCI respectively. With the APS rising from 39.22 to 157.77, the most pronounced association was detected between single NO exposure and MCI as the HR being 1.62 (95% CI: 1.3 to 1.63). However, we also found that, compared with other subtypes of cognitive impairment and dementia, vascular dementia only appeared to be influenced by NO and NO_2_ exposure, the HRs of which in the last quintile were 1.22 (95% CI: 1.03 to 1.45) and 1.23 (95% CI: 1.04 to 1.46) respectively. The detailed outcomes were available in Supplementary Table S6.

When it comes to the combined exposure to various air pollutants, compared with participants in the first quintile, the HRs of those in the last quintile for the air pollution-related cognitive impairments and dementia, including all-cause dementia, Alzheimer’s dementia, vascular dementia, and MCI, were 1.07 (95% CI: 1.04 to 1.09), 1.08 (95% CI: 1.04 to 1.12), 1.07 (95% CI: 1.02 to 1.13), and 1.19 (95% CI: 1.12 to 1.27) respectively. The detailed results were shown in Table 2.

**Table 2:** The HRs and 95% CIs for the association between combined air pollutants exposure and the risk of incident cognitive impairment and dementia, adjusted by potential confounders

	Q1 (39.22, 49.82)	Q2 (49.82, 54.61)	Q3 (54.61, 58.38)	Q4 (58.38, 63.09)	Q5 (63.09, 157.77)	HR, std	P for trend
**All-cause dementia**							
Case/person-years	1230/1,154,625	1384/1,150,136	1465/1,144,651	1459/1,142,135	1458/1,147,265		
Model1	Reference	1.15 (1.06–1.24)	1.26 (1.17–1.36)	1.34 (1.24–1.44)	1.45 (1.34–1.56)	1.13 (1.1–1.15)	2.71295E-25
Model2	Reference	1.08 (1–1.17)	1.15 (1.06–1.24)	1.19 (1.1–1.29)	1.26 (1.17–1.36)	1.08 (1.05–1.11)	2.46186E-10
Model3	Reference	1.07 (0.99–1.15)	1.13 (1.05–1.22)	1.16 (1.08–1.25)	1.21 (1.12–1.31)	1.07 (1.04–1.09)	1.02076E-07
**Alzheimer’s dementia**							
Case/person-years	530/1,156,231	598/1,151,669	581/1,146,771	590/1,144,482	628/1,149,591		
Model1	Reference	1.15 (1.02–1.29)	1.16 (1.03–1.3)	1.26 (1.12–1.41)	1.45 (1.29–1.63)	1.12 (1.09–1.16)	2.31653E-10
Model2	Reference	1.09 (0.97–1.22)	1.07 (0.95–1.2)	1.14 (1.01–1.28)	1.3 (1.15–1.46)	1.09 (1.05–1.13)	2.88284E-05
Model3	Reference	1.09 (0.97–1.22)	1.06 (0.95–1.2)	1.13 (1–1.27)	1.28 (1.14–1.44)	1.08 (1.04–1.12)	8.42853E-05
**Vascular dementia**							
Case/person-years	244/1,156,781	313/1,152,836	328/1,147,997	321/1,145,401	338/1,149,376		
Model1	Reference	1.3 (1.1–1.53)	1.43 (1.21–1.69)	1.48 (1.26–1.75)	1.68 (1.43–1.98)	1.16 (1.11–1.22)	3.39876E-10
Model2	Reference	1.21 (1.02–1.43)	1.27 (1.07–1.5)	1.29 (1.09–1.52)	1.41 (1.19–1.66)	1.1 (1.05–1.16)	0.000113222
Model3	Reference	1.18 (0.99–1.39)	1.22 (1.04–1.45)	1.21 (1.03–1.44)	1.3 (1.1–1.54)	1.07 (1.02–1.13)	0.004549959
**MCI**							
Case/person-years	126/1,156,762	143/1,152,707	153/1,148,090	192/1,145,912	230/1,149,732		
Model1	Reference	1.15 (0.91–1.46)	1.28 (1.01–1.62)	1.69 (1.35–2.11)	2.13 (1.72–2.65)	1.26 (1.19–1.34)	4.45858E-15
Model2	Reference	1.09 (0.86–1.39)	1.16 (0.92–1.47)	1.49 (1.19–1.88)	1.79 (1.44–2.24)	1.21 (1.13–1.28)	1.63128E-09
Model3	Reference	1.07 (0.84–1.36)	1.14 (0.9–1.44)	1.44 (1.15–1.81)	1.71 (1.37–2.14)	1.19 (1.12–1.27)	2.34471E-08

Model1 was adjusted for age and sex

Model2 was further adjusted for ethnicity (white/non-white), education (0–7 years, 8–10 years, 11–15 years, or 16- years), income(continuous), work(employed/unemployed), drinking status (never/past/current), and smoking status (never/past/current)

Model3 was additionally adjusted for BMI (continuous), SBP (continuous), and physical activity (MET-min/week).

Abbreviations: HR, hazard ratio; CI, confidence interval; Q1, the first quartile; Q2, the second quartile; Q3, the third quartile; Q4, the fourth quartile; Q5, the final quartile

**Fig. 1:**
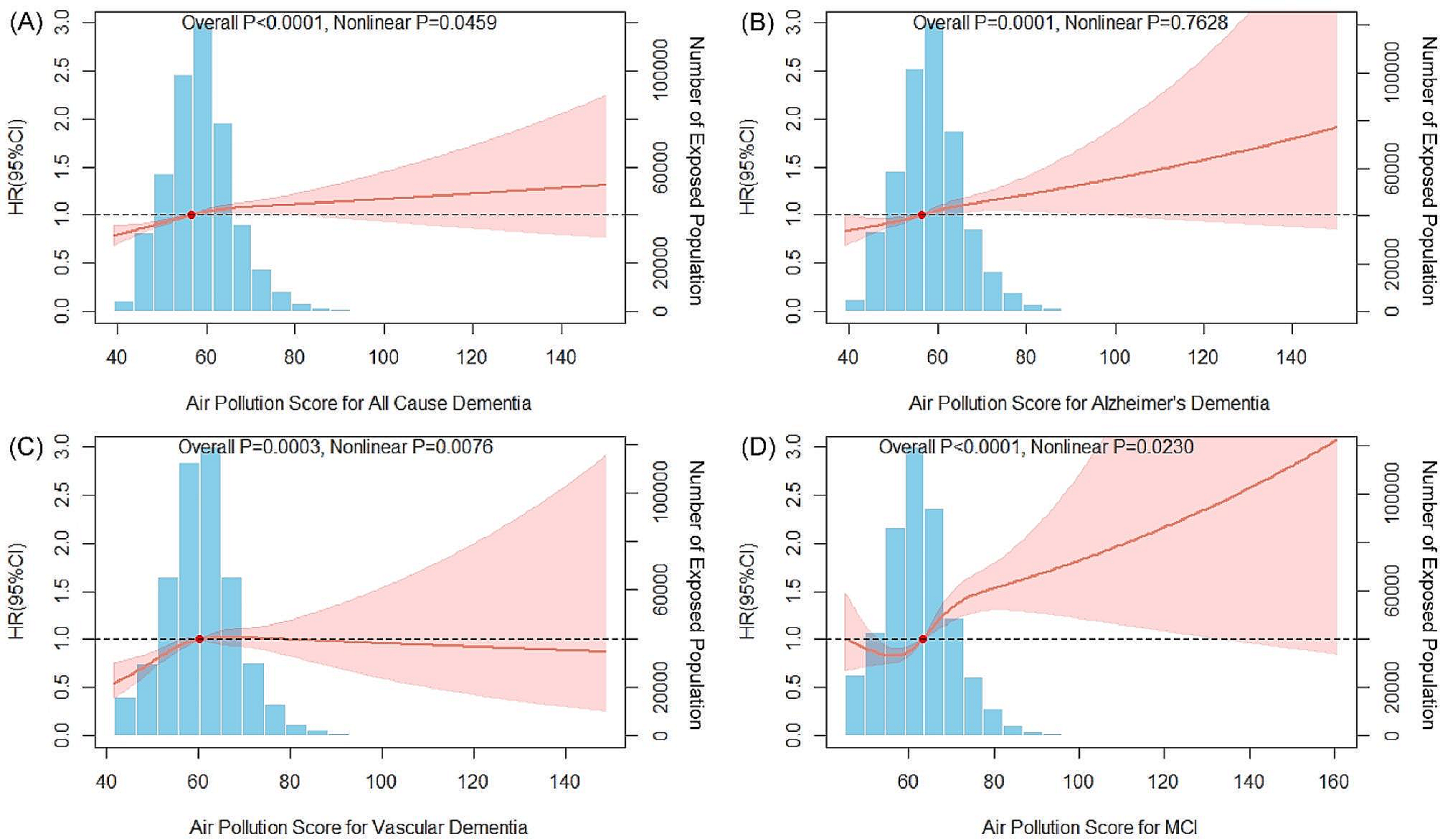
The restricted cubic spline curves of air pollution exposure and cognitive impairment. (**A**) All-cause dementia. (**B**) Alzheimer’s dementia. (**C**) Vascular dementia. (**D**) Mild cognitive impairment (MCI). Blue bars represent the distribution of the exposure levels in the entire population. The red solid line indicates HR and the shaded area indicates a 95% confidence interval

Although all four subtypes of cognitive impairment and dementia were proven to be statistically associated with combined air pollution exposure, yet only the incidence of Alzheimer’s dementia showed a significant linearity with the APS (P for nonlinear = 0.7628). The results were presented in Fig. 1. Regarding the single air pollutant exposure, the outcomes of which became diverse. Several linear relationships were found between the incident MCI and the exposure of NO_2_, PM_2.5_, PM_10_, and PM_2.5−10_, as well as the incident Alzheimer’s dementia and the exposure of NO, NO_2_, and PM_2.5_. However, with only NO_2_ and PM_10_ exposure being linear with pollution-induced all-cause dementia and vascular dementia respectively, the linearities of which were considered to be less pronounced compared with other two subtypes of cognitive impairment and dementia. The detailed results were displayed in Supplementary Figure S2, S3, S4, and S5.

### Effect modification by genetic predisposition

**Fig. 2:**
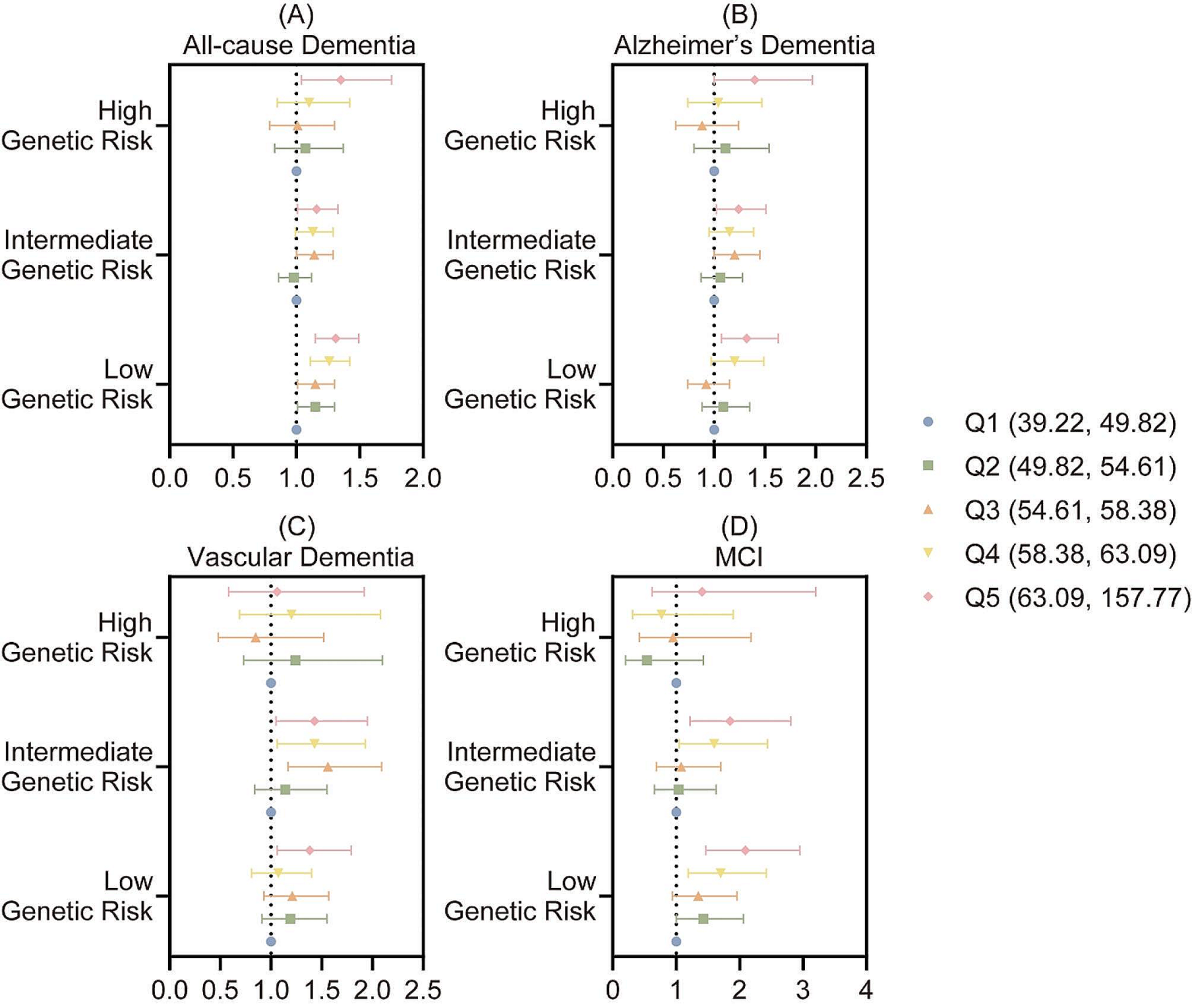
The associations between the genetic risk and cognitive impairment and dementia. (**A**) All-cause dementia. (**B**) Alzheimer’s dementia. (**C**) Vascular dementia. (**D**) Mild cognitive impairment (MCI)

In the current study, we did not observe significant interactions between the genetic risk and the air pollutants exposure on the incidence of cognitive decline. The outcomes suggested that the association between air pollution and cognitive impairment was stable and not likely to be influenced by participants’ genetic predisposition. However, researchers still noticed that participants in high genetic predisposition group might face a greater risk of air pollution induced cognitive impairment in general, particularly all-cause dementia, although the results were proven to be statistically insignificant. The detailed outcomes of combined exposure were presented in Fig. 2, while those of single air pollutant exposure were available in Supplementary Table S7.

### Effect modification by healthy lifestyle

In the aspect of healthy lifestyle, researchers found that it was likely to be a favourable factor in reducing the risk of air pollution-induced all-cause dementia and Alzheimer’s dementia, the HRs of which dropped from 1.34 (95% CI: 1.2 to 1.49) in the second quintile to 0.99 (95% CI: 0.84 to 1.17) in the last quintile and from 1.37 (95% CI: 1.16 to 1.63) in the second quintile to 1.07 (95% CI: 0.85 to 1.36) in the last quintile respectively. However, a healthy lifestyle was unable to effectively stop the development of vascular dementia caused by exposure to various air pollutants, regardless of combined or single. As for the incident MCI, we found that a healthy lifestyle was only statistically positive under the circumstances of single NO_2_ exposure, as the HR declined from 1.84 (95% CI: 1.34 to 2.52) in the second quintile to 1.45 (95% CI: 0.92 to 2.29) in the last quintile.

**Fig. 3:**
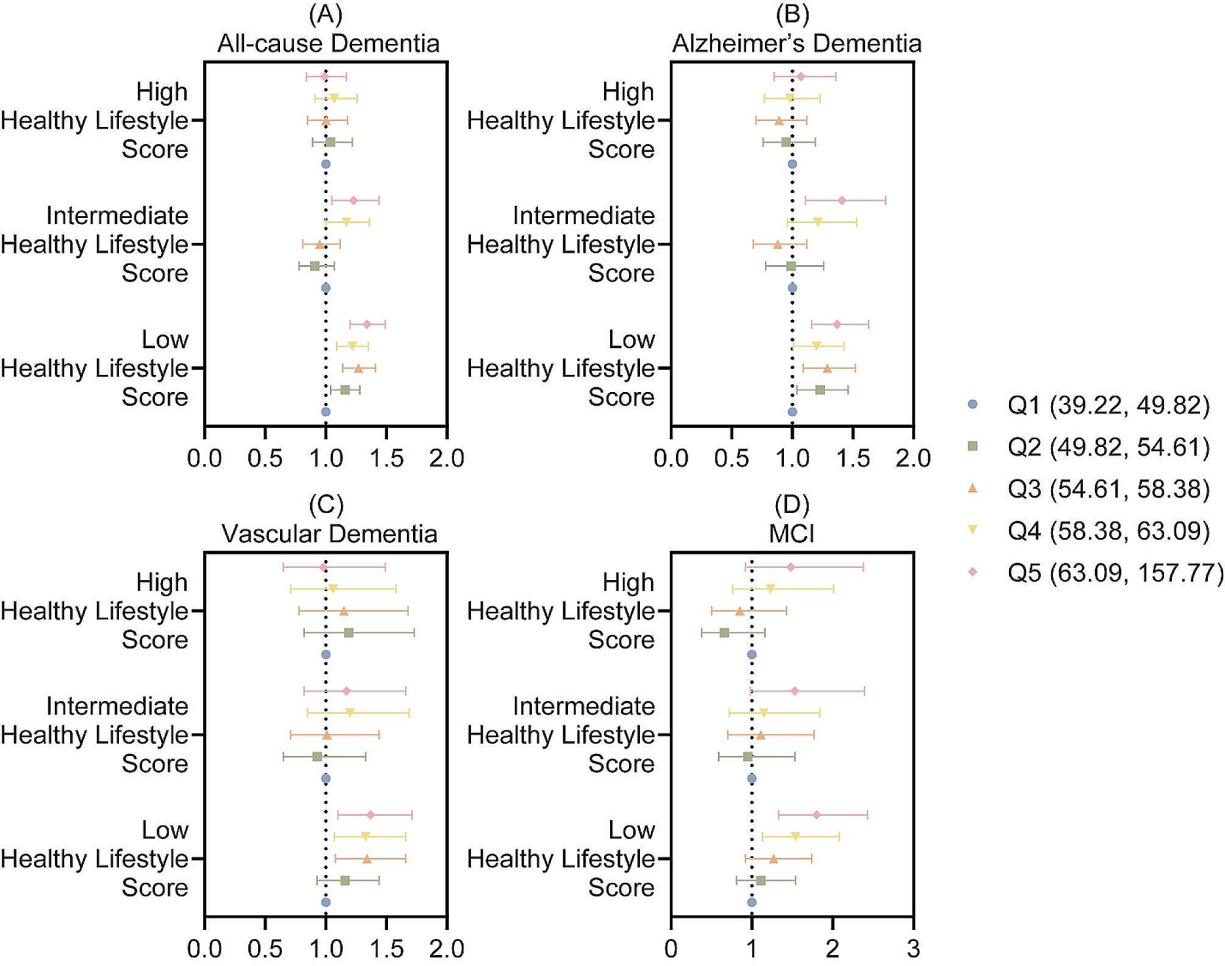
The associations between the healthy lifestyle and cognitive impairment and dementia. (**A**) All-cause dementia. (**B**) Alzheimer’s dementia. (**C**) Vascular dementia. (**D**) Mild cognitive impairment (MCI)

Although healthy lifestyle failed to show its statistical significance in most cognitive decline cases, with the HLS rising, research team did observe an uptrend in the incidence of cognitive dysfunction associated with air pollution exposure. The results of combined exposure were presented in Fig. 3, while those of single air pollutant exposure were in available Supplementary Table S8.

### Effect modification by other potential confounders

We found that participants with the previous history of CVD had a higher incidence of air pollution-induced Alzheimer’s dementia, the HR of which was 1.3 (95% CI: 0.95 to 1.79). In addition, combined with air pollutants, both younger age (< 60 years) and the previous history of hypertension turned into risk factors for the development of MCI, and the HRs were 1.7 (95% CI: 1.04 to 2.78) and 2.32 (95% CI: 1.59 to 3.39) respectively. Detailed results were provided in Supplementary Table S9.

### Sensitivity analysis

After excluding diagnosed cases within the first 2 years of follow-up, HRs of which were similar in magnitude to those obtained in the main results using the fully adjusted models. Another sensitivity analysis by restricting the participants’ follow-up durations to at least 5 years was also conducted. The outcomes showed only the association between joint air pollution exposure and vascular dementia became statistically insignificant with HR being 1.06 (95% CI: 1 to 1.12), suggesting that the short-term air pollution observation might interfere with the assessment of air pollution-induced vascular dementia. Moreover, the possibility that the results might be influenced by other mixed confounders, including age, sex, ethnicity, etc., was also excluded. The HRs were basically in line with those from the main results using the fully adjusted models. Overall, except the air pollution observation for less than 5 years may cast its influence on the long-term evaluation between air pollution and vascular dementia, all other results were confirmed to be robust and reliable. The details were presented in Supplementary Table S10, S11, and S12.

### Population attributable fraction (PAF)

The results of PAF showed that if the single air pollutant (NO_2_, NO, PM_10_, PM_2.5_, and PM_2.5−10_) exposed category were shifted into an unexposed category, 2.1%, 2.12%, 1.63%, 2.43%, and 0.79% of cognitive decline cases might be preventable, respectively. In another word, approximate 9.07% of which were potentially evitable if they were not under the combined exposure to various air pollutants. The outcomes were available in Table 3.

**Table 3:** The PAF for cognitive impairment risk factors in the UK Biobank

Exposure	PAF	Communality	Weighted PAF
NO_2_	12.22	88.59	2.1
NO	12.35	90.76	2.12
PM_10_	9.51	80.24	1.63
PM_2.5_	14.15	90.33	2.43
PM_2.5−10_	4.59	21.29	0.79
Combined exposure	42.97	-	-
Overall weighted	-	-	9.07

## Discussion

In this study based on the data from the UK Biobank prospective cohort, the long-term combined exposure to air pollutants consisting of PM_2.5_, PM_10_, PM_2.5−10_, NO_2_, and NO, which was presented in the form of an APS, was found to be associated with the increase in the incidence of cognitive impairment and dementia. In terms of the combined air pollution exposure, compared with the participants in the low exposure group, the risks of developing all-cause dementia, Alzheimer’s dementia, vascular dementia, and MCI in those exposed to the highest level of pollution rose by 7%, 8%, 7%, and 19% respectively, suggesting the existence of a synergistic effect between five individual air pollutants. When it comes to single pollutant exposure, except PM_2.5−10_, all other four individual air pollutants showed their connections with cognitive dysfunction. Moreover, our research team discovered that previous well-documented risk factors, such as genetic predisposition, turned out to be basically irrelevant to cognitive impairments caused by air pollution, while to which younger age, hypertension, and CVD were proven to be contributing factors. In terms of a healthy lifestyle, its ability to fight against the development of air pollution-induced cognitive decline was relatively limited.

Air pollution, as the largest environmental threat to today’s worldwide public health, has been strongly urged to be addressed through global collaboration [38]. In recent years, with mounting studies from low-income and middle-income countries being published, the research that focused on air pollution not only obtained an increase in quality but was also becoming more inclusive [39]. As a result, the ability of various ambient air pollutants to cast adverse effects on cognitive function was widely confirmed by researchers around the world. The findings of our current study were also consistent with previous investigations, that combined exposure to various air pollutants was responsible for cognitive decline in the general population. However, in terms of single air pollutant exposure, the outcomes of PM_2.5−10_ were not in line with the previous study [40], which might be the consequence of the lack of participants at that moment.

Hypotheses were proposed by several previous studies to explain the exact mechanism lying behind the association between air pollution exposure and cognitive decline. Animal experiments [41–43] found that inhaled PM, especially PM_2.5_, may directly reach the cerebrum by penetrating into the olfactory bulb, the frontal cortical, and subcortical areas, causing oxidative stress and inflammation. Further autopsy studies [44, 45] based on children and young adults living in Mexico City also supported this theory, pointing out the existence of connections between urban air pollution exposure, particulate deposition, and inflammation already present within the brain. In addition, air pollutant was also believed to place a burden on the cerebral proteostasis network, eventually leading to its collapse, disrupting the health of the proteome, and causing cell death and neurodegenerative disease [46]. However, in terms of the underlying mechanism between nitrogen oxides and cognitive impairment, it remained a complicated question. In fact, an early investigation [47] based on ischemic stroke mice models has already suggested that exposure to NO_2_ might act as a potential risk factor for the development of vascular dementia by inhibiting the expression of synaptophysin and postsynaptic density protein 95, two structural markers of synapses. Moreover, several recent studies [48, 49] also observed that NO_2_ exposure was associated with higher levels of brain dementia-related amyloid-β deposition and AD-like cortical atrophy in cognitively unimpaired individuals, causing poorer cognitive function among dementia-free population.

The genetic predisposition was widely recognized as a risk factor for the occurrence of cognitive impairment and dementia [14–19]. Former studies [50, 51], which aimed to explore the potential relationship between gene variants and the cognitive dysfunction associated with air pollution, have confirmed that APOE-ε4 carriers faced a greater risk of cognitive impairment when they were under exposure to ambient air pollutants. However, in this paper, it was noticed by researchers that the genetic risk, regardless of low, intermediate, or high, was basically unrelated to the air pollution-induced cognitive decline. Several conjectures were put forward by the research team to explain the discrepancy between the results of former and present studies. First, the participants of two previous studies were recruited from Manhattan, America, and Ruhr, Germany respectively. But the participants of the UK Biobank project were mainly living in the UK during the follow-up. Therefore, we could not exclude the possibility of inconsistent outcomes due to the difference in the geographic regions of the cohort populations. Second, similar to the explanation for discordant findings of PM_2.5−10_ exposure, the number of participants in previous studies was significantly smaller compared with that in the present study, which might suggest an increase in the risk of misjudgement caused by the limitation of included study population. While genetic predisposition might not influence the susceptibility to air pollution-related cognitive decline, adopting a healthy lifestyle could play a vital role in mitigating this risk.

A healthy lifestyle was found to be helpful in fighting against the development of pollution-related cognitive decline, although the correlations between them were not statistically significant. This result was consistent with that from previous studies [52, 53]. The reduction in the risk of cognitive impairment and dementia might be associated with the better prevention of hyperlipidaemia and diabetes attributed to a healthier lifestyle. Moreover, considering that the history of CVD and hypertension were highly likely to bring damage to the cerebrovascular system, the negative impact on cognitive impairment and dementia management caused by these two diseases appeared to be understandable. Yet in terms of a younger age may worsen the prevention of air pollution-related cognitive decline, unfortunately, we could not provide a specific explanation based on our current research finding, and further investigations are desperately needed in the future.

To our knowledge, this paper might be the first study to fully analyse and describe the associations between long-term air pollution exposure, genetic risk, lifestyle, and incident cognitive decline by using the latest findings from the UK Biobank. The measurements of combined air pollutants exposure and genetic predisposition were presented in the form of an APS and the genetic risk level for cognitive impairment, making the analysis more accurate and easier to perform. Compared with other previous studies that used date from the same UK Biobank database, our current work has included significantly greater study population (*n* = 460,872), and based on which we successfully observed that both individual and combined exposures to various air pollutants are hazardous to the cognitive function in human-beings. More interestingly, in addition to the association between air pollution exposure and cognitive damage, we also further explored the protentional modification effect by healthy lifestyle and genetic factor, pointing out that adopting a healthy and appropriate lifestyle can be used as a practical and novel approach to better combating the increasing and ongoing air pollution-related dementia and cognitive impairment worldwide. However, several potential limitations could not be ignored. First, most of the participants in the UK Biobank study were of European descent. As a result, whether the findings of our present study could be applied to other ethnicities across the world remained unknown, which called for more thoughtful investigations in the future. Second, despite the UK Biobank providing detailed and comprehensive air pollution information, other possible ambient air pollutants, such as polycyclic aromatic hydrocarbons (PAHs), O_3_, and SO_2_, were not included in this study. Meanwhile, given that the air pollution exposure could be of multiple origins, but the data on indoor and traffic-related air pollutants were unavailable. Third, only the APOE gene may be insufficient to fully estimate the genetic risk of cognitive decline, the results of which should be interpreted with caution. Fourth, the data on air pollution exposure during the follow-up were inaccessible to the research team. Finally, although typical covariates and risk factors were brought into consideration in this study, the risk of residual confounding still could not be completely excluded.

## Conclusion

Overall, in the current study, researchers found that both single and combined exposures to various air pollutants were associated with greater risks of cognitive impairment and dementia. However, the genetic predisposition, as a traditional and well-documented risk factor of cognitive decline, was unable to significantly modify the association between air pollution and cognitive impairment and dementia. Meanwhile, a healthy lifestyle was evaluated to be a partly effective means of lowering the incidence of cognitive dysfunction caused by air pollution exposure, while to which other potential confounders, including younger age, CVD, and hypertension, were proven to be unfavourable. It’s important to note that while a healthy lifestyle might help lower the risk, it may not entirely eliminate the impact of air pollution on cognitive health. Therefore, comprehensive efforts to address air pollution through environmental regulations and policies remain crucial for safeguarding cognitive well-being across populations.

### Electronic supplementary material

Below is the link to the electronic supplementary material.

Supplementary Material 1

## Data Availability

The data used for analysis are available from the corresponding author on reasonable request. The original dataset was acquired from the UK Biobank (https://www.ukbiobank.ac.uk/) and shall not be used for any commercial purpose without permission.

## Abbreviations

PM: Particulate matter
PM_2.5_: Particulate matter with diameters ≤ 2.5 μm
PM_10_: Particulate matter with diameters ≤ 10 μm
PM_2.5−10_: Particulate matter with diameters between 2.5 and 10 μm
NO and NO_2_: Nitrogen oxides
APS: Air pollution score
HLS: Healthy lifestyle score
HR: Hazard ratio
CI: Confidence interval
MCI: Mild cognitive impairment
AIDS: Acquired immune deficiency syndrome
PAF: Population attributable fraction
ICD-10: International Classification of Diseases, 10th revision
GWAS: Genome-wide association study
ESCAPE: European Study of Cohorts for Air Pollution Effects
APOE: Apolipoprotein E
BMI: Body mass index
WHR: Waist-to-hip ratio
IPAQ: International Physical Activity Questionnaire
MET: Metabolic equivalent task
WC: Waist circumference
HC: Hip circumference
TDI: Townsend deprivation index
SBP: Systolic blood pressure
CAD: Coronary artery disease
CVD: Cardiovascular disease
RR: Risk ratio
PAHs: Polycyclic aromatic hydrocarbons

## References

[1] White A, Kanninen K, Malm T, Schins R (2020). Editorial: air pollution and brain health. Neurochem Int.

[2] Landrigan PJ, Fuller R, Acosta NJR, Adeyi O, Arnold R, Basu NN (2018). The Lancet Commission on pollution and health. Lancet.

[3] Prince M, Bryce R, Albanese E, Wimo A, Ribeiro W, Ferri CP (2013). The global prevalence of dementia: a systematic review and metaanalysis. Alzheimers Dement.

[4] Hahad O, Lelieveld J, Birklein F, Lieb K, Daiber A, Münzel T. Ambient air Pollution increases the risk of Cerebrovascular and Neuropsychiatric disorders through induction of inflammation and oxidative stress. Int J Mol Sci. 2020; 21(12). 10.3390/ijms21124306 PMC7352229 32560306

[5] Delgado-Saborit JM, Guercio V, Gowers AM, Shaddick G, Fox NC, Love S (2021). A critical review of the epidemiological evidence of effects of air pollution on dementia, cognitive function and cognitive decline in adult population. Sci Total Environ.

[6] Kivipelto M, Mangialasche F, Ngandu T (2018). Lifestyle interventions to prevent cognitive impairment, dementia and Alzheimer disease. Nat Rev Neurol.

[7] Loy CT, Schofield PR, Turner AM, Kwok JB (2014). Genetics of dementia. Lancet.

[8] Sudlow C, Gallacher J, Allen N, Beral V, Burton P, Danesh J (2015). UK biobank: an open access resource for identifying the causes of a wide range of complex diseases of middle and old age. PLoS Med.

[9] Rusk N (2018). The UK Biobank. Nat Methods.

[10] Wong PY, Lee HY, Chen YC, Zeng YT, Chern YR, Chen NT (2021). Using a land use regression model with machine learning to estimate ground level PM(2.5). Environ Pollut.

[11] Eeftens M, Beelen R, de Hoogh K, Bellander T, Cesaroni G, Cirach M (2012). Development of Land Use Regression models for PM(2.5), PM(2.5) absorbance, PM(10) and PM(coarse) in 20 European study areas; results of the ESCAPE project. Environ Sci Technol.

[12] Beelen R, Hoek G, Vienneau D, Eeftens M, Dimakopoulou K, Pedeli X (2013). Development of NO2 and NOx land use regression models for estimating air pollution exposure in 36 study areas in Europe– The ESCAPE project. Atmos Environ.

[13] Wang M, Zhou T, Song Y, Li X, Ma H, Hu Y (2021). Joint exposure to various ambient air pollutants and incident heart failure: a prospective analysis in UK Biobank. Eur Heart J.

[14] Bellenguez C, Küçükali F, Jansen IE, Kleineidam L, Moreno-Grau S, Amin N (2022). New insights into the genetic etiology of Alzheimer’s disease and related dementias. Nat Genet.

[15] Gouveia C, Gibbons E, Dehghani N, Eapen J, Guerreiro R, Bras J (2022). Genome-wide association of polygenic risk extremes for Alzheimer’s disease in the UK Biobank. Sci Rep.

[16] Jansen IE, Savage JE, Watanabe K, Bryois J, Williams DM, Steinberg S (2019). Genome-wide meta-analysis identifies new loci and functional pathways influencing Alzheimer’s disease risk. Nat Genet.

[17] Kunkle BW, Grenier-Boley B, Sims R, Bis JC, Damotte V, Naj AC (2019). Genetic meta-analysis of diagnosed Alzheimer’s disease identifies new risk loci and implicates Aβ, tau, immunity and lipid processing. Nat Genet.

[18] Schwartzentruber J, Cooper S, Liu JZ, Barrio-Hernandez I, Bello E, Kumasaka N (2021). Genome-wide meta-analysis, fine-mapping and integrative prioritization implicate new Alzheimer’s disease risk genes. Nat Genet.

[19] Wightman DP, Jansen IE, Savage JE, Shadrin AA, Bahrami S, Rongve A et al. Largest GWAS (N = 1,126,563) of Alzheimer’s Disease implicates Microglia and Immune cells. medRxiv. 2020:2020.11.20.20235275.

[20] Bycroft C, Freeman C, Petkova D, Band G, Elliott LT, Sharp K et al. Genome-wide genetic data on ~ 500,000 UK Biobank participants. bioRxiv. 2017:166298.

[21] Ikram MA, Bersano A, Manso-Calderón R, Jia JP, Schmidt H, Middleton L (2017). Genetics of vascular dementia - review from the ICVD working group. BMC Med.

[22] Martens YA, Zhao N, Liu CC, Kanekiyo T, Yang AJ, Goate AM (2022). ApoE Cascade Hypothesis in the pathogenesis of Alzheimer’s disease and related dementias. Neuron.

[23] Aune D, Sen A, Prasad M, Norat T, Janszky I, Tonstad S (2016). BMI and all cause mortality: systematic review and non-linear dose-response meta-analysis of 230 cohort studies with 3.74 million deaths among 30.3 million participants. BMJ.

[24] Bhaskaran K, Dos-Santos-Silva I, Leon DA, Douglas IJ, Smeeth L (2018). Association of BMI with overall and cause-specific mortality: a population-based cohort study of 3·6 million adults in the UK. Lancet Diabetes Endocrinol.

[25] Flegal KM, Kit BK, Orpana H, Graubard BI (2013). Association of all-cause mortality with overweight and obesity using standard body mass index categories: a systematic review and meta-analysis. JAMA.

[26] Global BMIMC, Di Angelantonio E, Bhupathiraju Sh N, Wormser D, Gao P, Kaptoge S (2016). Body-mass index and all-cause mortality: individual-participant-data meta-analysis of 239 prospective studies in four continents. Lancet.

[27] Peter RS, Mayer B, Concin H, Nagel G (2015). The effect of age on the shape of the BMI-mortality relation and BMI associated with minimum all-cause mortality in a large Austrian cohort. Int J Obes (Lond).

[28] Craig CL, Marshall AL, Sjöström M, Bauman AE, Booth ML, Ainsworth BE (2003). International physical activity questionnaire: 12-country reliability and validity. Med Sci Sports Exerc.

[29] Lee PH, Macfarlane DJ, Lam TH, Stewart SM (2011). Validity of the International Physical Activity Questionnaire Short Form (IPAQ-SF): a systematic review. Int J Behav Nutr Phys Act.

[30] Elliott AD, Linz D, Mishima R, Kadhim K, Gallagher C, Middeldorp ME (2020). Association between physical activity and risk of incident arrhythmias in 402 406 individuals: evidence from the UK Biobank cohort. Eur Heart J.

[31] Choi J, Jia G, Wen W, Shu XO, Zheng W (2021). Healthy lifestyles, genetic modifiers, and colorectal cancer risk: a prospective cohort study in the UK Biobank. Am J Clin Nutr.

[32] Ho FK, Gray SR, Welsh P, Petermann-Rocha F, Foster H, Waddell H (2020). Associations of fat and carbohydrate intake with cardiovascular disease and mortality: prospective cohort study of UK Biobank participants. BMJ.

[33] Townsend P, Deprivation (1987). J Social Policy.

[34] Levin ML (1953). The occurrence of lung cancer in man. Acta Unio Int Contra Cancrum.

[35] Mansournia MA, Altman DG (2018). Population attributable fraction. BMJ.

[36] Shield KD, Parkin DM, Whiteman DC, Rehm J, Viallon V, Micallef CM (2016). Population Attributable and preventable fractions: Cancer risk factor surveillance, and Cancer Policy Projection. Curr Epidemiol Rep.

[37] Mukadam N, Sommerlad A, Huntley J, Livingston G (2019). Population attributable fractions for risk factors for dementia in low-income and middle-income countries: an analysis using cross-sectional survey data. Lancet Glob Health.

[38] Fuller R, Landrigan PJ, Balakrishnan K, Bathan G, Bose-O’Reilly S, Brauer M (2022). Pollution and health: a progress update. Lancet Planet Health.

[39] The Lancet Respiratory M (2021). Air pollution-time to address the silent killer. Lancet Respir Med.

[40] Chandra M, Rai CB, Kumari N, Sandhu VK, Chandra K, Krishna M et al. Air Pollution and Cognitive Impairment across the life course in humans: a systematic review with specific focus on Income Level of Study Area. Int J Environ Res Public Health. 2022; 19(3). 10.3390/ijerph19031405 PMC8835599 35162428

[41] Nemmar A, Vanbilloen H, Hoylaerts MF, Hoet PH, Verbruggen A, Nemery B (2001). Passage of intratracheally instilled ultrafine particles from the lung into the systemic circulation in hamster. Am J Respir Crit Care Med.

[42] Elder A, Gelein R, Silva V, Feikert T, Opanashuk L, Carter J (2006). Translocation of inhaled ultrafine manganese oxide particles to the central nervous system. Environ Health Perspect.

[43] Cheng H, Saffari A, Sioutas C, Forman HJ, Morgan TE, Finch CE (2016). Nanoscale Particulate Matter from Urban Traffic rapidly induces oxidative stress and inflammation in olfactory epithelium with concomitant effects on Brain. Environ Health Perspect.

[44] Calderón-Garcidueñas L, Franco-Lira M, Henríquez-Roldán C, Osnaya N, González-Maciel A, Reynoso-Robles R (2010). Urban air pollution: influences on olfactory function and pathology in exposed children and young adults. Exp Toxicol Pathol.

[45] Calderón-Garcidueñas L, Solt AC, Henríquez-Roldán C, Torres-Jardón R, Nuse B, Herritt L (2008). Long-term air pollution exposure is associated with neuroinflammation, an altered innate immune response, disruption of the blood-brain barrier, ultrafine particulate deposition, and accumulation of amyloid beta-42 and alpha-synuclein in children and young adults. Toxicol Pathol.

[46] Kikis EA (2020). The proteostatic effects of traffic-derived air pollution on Alzheimer’s disease risk. Open Biol.

[47] Li H, Xin X (2013). Nitrogen dioxide (NO(2)) pollution as a potential risk factor for developing vascular dementia and its synaptic mechanisms. Chemosphere.

[48] Alemany S, Crous-Bou M, Vilor-Tejedor N, Milà-Alomà M, Suárez-Calvet M, Salvadó G (2021). Associations between air pollution and biomarkers of Alzheimer’s disease in cognitively unimpaired individuals. Environ Int.

[49] Cho J, Jang H, Park H, Noh Y, Sohn J, Koh SB (2023). Alzheimer’s disease-like cortical atrophy mediates the effect of air pollution on global cognitive function. Environ Int.

[50] Kulick ER, Elkind MSV, Boehme AK, Joyce NR, Schupf N, Kaufman JD (2020). Long-term exposure to ambient air pollution, APOE-ε4 status, and cognitive decline in a cohort of older adults in northern Manhattan. Environ Int.

[51] Schikowski T, Vossoughi M, Vierkötter A, Schulte T, Teichert T, Sugiri D (2015). Association of air pollution with cognitive functions and its modification by APOE gene variants in elderly women. Environ Res.

[52] Dhana K, Franco OH, Ritz EM, Ford CN, Desai P, Krueger KR (2022). Healthy lifestyle and life expectancy with and without Alzheimer’s dementia: population based cohort study. BMJ.

[53] Dhana K, Evans DA, Rajan KB, Bennett DA, Morris MC (2020). Healthy lifestyle and the risk of Alzheimer dementia: findings from 2 longitudinal studies. Neurology.

