# Supplementary material for: Association of long-term exposure to various ambient air pollutants, lifestyle, and genetic predisposition with incident cognitive impairment and dementia
Source: BMC Public Health. 2024 Jan 15;24:179. doi: 10.1186/s12889-024-17702-y (PMC10788974; doi:10.1186/s12889-024-17702-y)
Supplement: Supplementary file 1 — Supplementary Material 1 [file 12889_2024_17702_MOESM1_ESM.docx]

**UK Biobank**1 Study across 22 centers 502,412 Participants

502,149 Eligible participants with no dementia or MCI disease

460,872 Included in the analysis

Excluded with reasons:

Lacked the data on PM_2.5_, PM_10_, PM_2.5-10_, NO_2_, and NO exposure

**Supplementary Figure S1. Flow chart of study participants**

**Supplementary Table S1. Definitions and sources of information for dementia in the UK Biobank**

| Disease | Number of cases | ICD-10 diagnosis | UK Biobank data code |
| --- | --- | --- | --- |
| All cause Dementia | 6996 | G30, F01, G20. | 42018^a^ |
| Alzheimer's Dementia | 2927 | G30 | 42020 |
| Vascular Dementia | 1544 | F01 | 42022 |
| MCI | 844 | F06.7 | 41270 |
| ICD: International Classification of Disease.  ^a^ 42018 is the data code used in UK Biobank: All-cause dementia illness code | | | |

**Supplementary Table S2. Baseline characteristics of the UK Biobank participants (N=460,872) with Alzheimer's dementia in accordance with the quintiles of air pollution score**

|  | Air Pollution Score | | | | |
| --- | --- | --- | --- | --- | --- |
|  | Q1 (39.22, 49.82) | Q2 (49.82, 54.61) | Q3 (49.82, 54.61) | Q4 (58.38, 63.09) | Q5 (63.09, 157.77) |
| Follow-up duration, years | 12.5 (1.7) | 12.5 (1.8) | 12.4 (1.8) | 12.4 (1.8) | 12.5 (1.9) |
| Age, years | 57.8 (7.8) | 57.6 (8) | 57.3 (8.1) | 56.7 (8.2) | 56 (8.3) |
| Sex, n (%) |  |  |  |  |  |
| Male | 41959 (45.5) | 42012 (45.6) | 41888 (45.4) | 42042 (45.6) | 42586 (46.2) |
| Female | 50216 (54.5) | 50162 (54.4) | 50287 (54.6) | 50132 (54.4) | 49588 (53.8) |
| Ethnicity, n (%) |  |  |  |  |  |
| White | 90542 (98.2) | 89138 (96.7) | 87588 (95) | 84668 (91.9) | 80519 (87.4) |
| Non-white | 1633 (1.8) | 3036 (3.3) | 4587 (5) | 7506 (8.1) | 11655 (12.6) |
| TDI | -2.9 (2) | -2.4 (2.5) | -1.8 (2.7) | -0.8 (2.8) | 1.1 (3.3) |
| BMI, kg/m^2^ | 27.1 (4.5) | 27.4 (4.6) | 27.5 (4.8) | 27.6 (4.9) | 27.6 (5.1) |
| MET | 2710.4 (2723.3) | 2645.2 (2684.9) | 2661.9 (2720.6) | 2680.9 (2757.7) | 2640.7 (2725.4) |
| SBP, mmHg | 140.3 (21.3) | 139.8 (21.2) | 139.5 (21.3) | 139.3 (21.9) | 138.4 (22.5) |
| DBP, mmHg | 83.5 (13) | 83.4 (13) | 83.4 (13.1) | 83.7 (13.7) | 83.6 (14.5) |
| NO, μg/m^3^ | 26.9 (4.6) | 36.4 (4) | 42.3 (4) | 48.6 (4.6) | 65.9 (17.3) |
| NO_2_, μg/m^3^ | 17.2 (2.7) | 22.7 (2.4) | 26.3 (2.5) | 29.9 (2.6) | 37.2 (6.3) |
| PM_2.5_, μg/m^3^ | 8.7 (0.4) | 9.5 (0.3) | 9.9 (0.3) | 10.4 (0.4) | 11.5 (0.9) |
| PM_10_, μg/m^3^ | 14.3 (1.6) | 15.8 (1.3) | 16.4 (1.3) | 16.9 (1.5) | 17.8 (1.8) |
| PM_2.5-10_, μg/m^3^ | 6.2 (0.8) | 6.2 (0.8) | 6.3 (0.8) | 6.5 (0.9) | 6.9 (1) |
| Air Pollution Score | 45.3 (3) | 52.2 (1.3) | 56.3 (1.1) | 60.2 (1.3) | 69.4 (7.1) |
| Employment, n (%) |  |  |  |  |  |
| Yes | 51609 (56) | 51671 (56.1) | 52440 (56.9) | 54257 (58.9) | 55829 (60.6) |
| No | 40566 (44) | 40503 (43.9) | 39735 (43.1) | 37917 (41.1) | 36345 (39.4) |
| Education, n (%) |  |  |  |  |  |
| ≤7 years | 12154 (13.2) | 15204 (16.5) | 16876 (18.3) | 17804 (19.3) | 17709 (19.2) |
| 8~10 years | 15227 (16.5) | 16841 (18.3) | 17161 (18.6) | 16443 (17.8) | 14268 (15.5) |
| 11~15 years | 18179 (19.7) | 17196 (18.7) | 16330 (17.7) | 15410 (16.7) | 14155 (15.4) |
| ≥16 years | 46615 (50.6) | 42933 (46.6) | 41808 (45.4) | 42517 (46.1) | 46042 (50) |
| Income, n (%) |  |  |  |  |  |
| <£18,000 | 16046 (17.4) | 20656 (22.4) | 23457 (25.4) | 25878 (28.1) | 29499 (32) |
| £18,000~£52,000 | 48023 (52.1) | 48971 (53.1) | 48127 (52.2) | 46595 (50.6) | 42445 (46) |
| £52,000~£100,000 | 21592 (23.4) | 18327 (19.9) | 16942 (18.4) | 16035 (17.4) | 15047 (16.3) |
| >£100,000 | 6514 (7.1) | 4220 (4.6) | 3649 (4) | 3666 (4) | 5183 (5.6) |
| Smoke, n (%) |  |  |  |  |  |
| Never | 53433 (58) | 52047 (56.5) | 50810 (55.1) | 49887 (54.1) | 46247 (50.2) |
| Previous | 31999 (34.7) | 32210 (34.9) | 32157 (34.9) | 31747 (34.4) | 32268 (35) |
| Current | 6743 (7.3) | 7917 (8.6) | 9208 (10) | 10540 (11.4) | 13659 (14.8) |
| Drink, n (%) |  |  |  |  |  |
| Never | 2694 (2.9) | 3475 (3.8) | 4290 (4.7) | 4962 (5.4) | 6046 (6.6) |
| Previous | 2482 (2.7) | 2920 (3.2) | 3265 (3.5) | 3550 (3.9) | 4296 (4.7) |
| Current | 86999 (94.4) | 85779 (93.1) | 84620 (91.8) | 83662 (90.8) | 81832 (88.8) |
| Hypertension, n (%) | 23676 (25.7) | 25184 (27.3) | 25721 (27.9) | 25894 (28.1) | 25640 (27.8) |
| Diabetes, n (%) | 3648 (4) | 4466 (4.8) | 4794 (5.2) | 5341 (5.8) | 5569 (6) |
| CVD, n (%) | 12742 (13.8) | 13860 (15) | 14260 (15.5) | 14471 (15.7) | 14650 (15.9) |
| CAD, n (%) | 10380 (11.3) | 11470 (12.4) | 11821 (12.8) | 12021 (13) | 12032 (13.1) |
| Stroke, n (%) | 3286 (3.6) | 3520 (3.8) | 3626 (3.9) | 3653 (4) | 3939 (4.3) |

**Supplementary Table S3. Baseline characteristics of the UK Biobank participants (N=460,872) with vascular dementia in accordance with the quintiles of air pollution score**

|  | Air Pollution Score | | | | |
| --- | --- | --- | --- | --- | --- |
|  | Q1 (39.22, 49.82) | Q2 (49.82, 54.61) | Q3 (49.82, 54.61) | Q4 (58.38, 63.09) | Q5 (63.09, 157.77) |
| Follow-up duration, years | 12.5 (1.7) | 12.5 (1.8) | 12.5 (1.8) | 12.4 (1.8) | 12.5 (1.9) |
| Age, years | 57.7 (7.8) | 57.6 (8) | 57.3 (8.1) | 56.7 (8.2) | 56.1 (8.3) |
| Sex, n (%) |  |  |  |  |  |
| Male | 41991 (45.6) | 41987 (45.6) | 41902 (45.5) | 42028 (45.6) | 42579 (46.2) |
| Female | 50184 (54.4) | 50187 (54.4) | 50273 (54.5) | 50146 (54.4) | 49595 (53.8) |
| Ethnicity, n (%) |  |  |  |  |  |
| White | 90475 (98.2) | 89166 (96.7) | 87733 (95.2) | 84545 (91.7) | 80536 (87.4) |
| Non-white | 1700 (1.8) | 3008 (3.3) | 4442 (4.8) | 7629 (8.3) | 11638 (12.6) |
| TDI | -2.8 (2.1) | -2.3 (2.5) | -1.7 (2.7) | -0.8 (2.9) | 1 (3.4) |
| BMI, kg/m^2^ | 27.1 (4.5) | 27.4 (4.7) | 27.5 (4.8) | 27.6 (4.9) | 27.6 (5.1) |
| MET | 2714.7 (2729.5) | 2649.5 (2690.1) | 2666.8 (2718.9) | 2667.9 (2748.2) | 2640.2 (2725.2) |
| SBP, mmHg | 140.3 (21.3) | 139.9 (21.3) | 139.5 (21.3) | 139.2 (22) | 138.3 (22.3) |
| DBP, mmHg | 83.6 (13) | 83.4 (13) | 83.4 (13.1) | 83.7 (13.8) | 83.5 (14.3) |
| NO, μg/m^3^ | 27.4 (5) | 36.7 (4.8) | 42.6 (5) | 48.6 (6) | 64.8 (18.3) |
| NO_2_, μg/m^3^ | 17.5 (2.9) | 22.8 (2.7) | 26.4 (2.9) | 29.9 (3.3) | 36.6 (6.9) |
| PM_2.5_, μg/m^3^ | 8.7 (0.4) | 9.5 (0.4) | 10 (0.4) | 10.4 (0.5) | 11.4 (0.9) |
| PM_10_, μg/m^3^ | 14.1 (1.3) | 15.7 (0.9) | 16.3 (1) | 16.9 (1.3) | 18.2 (1.9) |
| PM_2.5-10_, μg/m^3^ | 6 (0.6) | 6.1 (0.7) | 6.2 (0.7) | 6.5 (0.9) | 7.2 (1.1) |
| Air Pollution Score | 49.2 (3.5) | 56.3 (1.3) | 60.2 (1) | 64.1 (1.3) | 72.6 (6.5) |
| Employment, n (%) |  |  |  |  |  |
| Yes | 51641 (56) | 51611 (56) | 52588 (57.1) | 54329 (58.9) | 55637 (60.4) |
| No | 40534 (44) | 40563 (44) | 39587 (42.9) | 37845 (41.1) | 36537 (39.6) |
| Education, n (%) |  |  |  |  |  |
| ≤7 years | 12421 (13.5) | 15278 (16.6) | 16954 (18.4) | 17476 (19) | 17618 (19.1) |
| 8~10 years | 15385 (16.7) | 16800 (18.2) | 16906 (18.3) | 16298 (17.7) | 14551 (15.8) |
| 11~15 years | 18050 (19.6) | 17262 (18.7) | 16291 (17.7) | 15341 (16.6) | 14326 (15.5) |
| ≥16 years | 46319 (50.3) | 42834 (46.5) | 42024 (45.6) | 43059 (46.7) | 45679 (49.6) |
| Income, n (%) |  |  |  |  |  |
| <£18,000 | 16368 (17.8) | 20865 (22.6) | 23607 (25.6) | 25593 (27.8) | 29103 (31.6) |
| £18,000~£52,000 | 48075 (52.2) | 48830 (53) | 48027 (52.1) | 46527 (50.5) | 42702 (46.3) |
| £52,000~£100,000 | 21346 (23.2) | 18240 (19.8) | 16962 (18.4) | 16256 (17.6) | 15139 (16.4) |
| >£100,000 | 6386 (6.9) | 4239 (4.6) | 3579 (3.9) | 3798 (4.1) | 5230 (5.7) |
| Smoke, n (%) |  |  |  |  |  |
| Never | 53435 (58) | 51905 (56.3) | 50785 (55.1) | 49603 (53.8) | 46696 (50.7) |
| Previous | 31937 (34.6) | 32301 (35) | 32099 (34.8) | 31847 (34.6) | 32197 (34.9) |
| Current | 6803 (7.4) | 7968 (8.6) | 9291 (10.1) | 10724 (11.6) | 13281 (14.4) |
| Drink, n (%) |  |  |  |  |  |
| Never | 2775 (3) | 3493 (3.8) | 4186 (4.5) | 4956 (5.4) | 6057 (6.6) |
| Previous | 2515 (2.7) | 2930 (3.2) | 3241 (3.5) | 3552 (3.9) | 4275 (4.6) |
| Current | 86885 (94.3) | 85751 (93) | 84748 (91.9) | 83666 (90.8) | 81842 (88.8) |
| Hypertension, n (%) | 23740 (25.8) | 25314 (27.5) | 25554 (27.7) | 25817 (28) | 25690 (27.9) |
| Diabetes, n (%) | 3706 (4) | 4480 (4.9) | 4791 (5.2) | 5193 (5.6) | 5648 (6.1) |
| CVD, n (%) | 12850 (13.9) | 13838 (15) | 14272 (15.5) | 14461 (15.7) | 14562 (15.8) |
| CAD, n (%) | 10491 (11.4) | 11478 (12.5) | 11771 (12.8) | 12016 (13) | 11968 (13) |
| Stroke, n (%) | 3278 (3.6) | 3516 (3.8) | 3686 (4) | 3629 (3.9) | 3915 (4.2) |

**Supplementary Table S4. Baseline characteristics of the UK Biobank participants (N=460,872) with MCI in accordance with the quintiles of air pollution score**

|  | Air Pollution Score | | | | |
| --- | --- | --- | --- | --- | --- |
|  | Q1 (39.22, 49.82) | Q2 (49.82, 54.61) | Q3 (49.82, 54.61) | Q4 (58.38, 63.09) | Q5 (63.09, 157.77) |
| Follow-up duration, years | 12.5 (1.7) | 12.5 (1.8) | 12.5 (1.8) | 12.4 (1.8) | 12.5 (1.9) |
| Age, years | 57.7 (7.8) | 57.6 (8) | 57.2 (8.1) | 56.7 (8.2) | 56.1 (8.3) |
| Sex, n (%) |  |  |  |  |  |
| Male | 41958 (45.5) | 41927 (45.5) | 41992 (45.6) | 41999 (45.6) | 42611 (46.2) |
| Female | 50217 (54.5) | 50247 (54.5) | 50183 (54.4) | 50175 (54.4) | 49563 (53.8) |
| Ethnicity, n (%) |  |  |  |  |  |
| White | 90489 (98.2) | 89140 (96.7) | 87659 (95.1) | 84528 (91.7) | 80639 (87.5) |
| Non-white | 1686 (1.8) | 3034 (3.3) | 4516 (4.9) | 7646 (8.3) | 11535 (12.5) |
| TDI | -2.8 (2) | -2.3 (2.5) | -1.7 (2.7) | -0.8 (2.9) | 1 (3.4) |
| BMI, kg/m^2^ | 27.1 (4.5) | 27.4 (4.7) | 27.5 (4.8) | 27.6 (4.9) | 27.6 (5.1) |
| MET | 2709.2 (2720.6) | 2648.9 (2692.9) | 2663.2 (2715.6) | 2674.3 (2754.2) | 2643.5 (2728.8) |
| SBP, mmHg | 140.2 (21.3) | 139.8 (21.2) | 139.6 (21.3) | 139.2 (22) | 138.3 (22.3) |
| DBP, mmHg | 83.5 (13) | 83.4 (12.9) | 83.4 (13.1) | 83.7 (13.8) | 83.5 (14.3) |
| NO, μg/m^3^ | 27.4 (4.8) | 36.6 (4.7) | 42.6 (4.8) | 48.6 (5.9) | 64.9 (18.2) |
| NO_2_, μg/m^3^ | 17.5 (2.9) | 22.8 (2.7) | 26.5 (2.9) | 29.9 (3.3) | 36.6 (6.9) |
| PM_2.5_, μg/m^3^ | 8.7 (0.4) | 9.5 (0.4) | 10 (0.4) | 10.4 (0.5) | 11.4 (0.9) |
| PM_10_, μg/m^3^ | 14.1 (1.3) | 15.7 (1) | 16.3 (1.1) | 16.9 (1.4) | 18.2 (1.9) |
| PM_2.5-10_, μg/m^3^ | 6 (0.5) | 6.2 (0.7) | 6.3 (0.7) | 6.5 (0.9) | 7.2 (1.1) |
| Air Pollution Score | 52.2 (3.3) | 59.2 (1.3) | 63.2 (1.1) | 67.4 (1.4) | 76.8 (7.2) |
| Employment, n (%) |  |  |  |  |  |
| Yes | 51544 (55.9) | 51653 (56) | 52611 (57.1) | 54419 (59) | 55579 (60.3) |
| No | 40631 (44.1) | 40521 (44) | 39564 (42.9) | 37755 (41) | 36595 (39.7) |
| Education, n (%) |  |  |  |  |  |
| ≤7 years | 12286 (13.3) | 15254 (16.5) | 16975 (18.4) | 17504 (19) | 17728 (19.2) |
| 8~10 years | 15380 (16.7) | 16755 (18.2) | 16897 (18.3) | 16333 (17.7) | 14575 (15.8) |
| 11~15 years | 18122 (19.7) | 17222 (18.7) | 16250 (17.6) | 15391 (16.7) | 14285 (15.5) |
| ≥16 years | 46387 (50.3) | 42943 (46.6) | 42053 (45.6) | 42946 (46.6) | 45586 (49.5) |
| Income, n (%) |  |  |  |  |  |
| <£18,000 | 16289 (17.7) | 20688 (22.4) | 23730 (25.7) | 25607 (27.8) | 29222 (31.7) |
| £18,000~£52,000 | 48135 (52.2) | 48790 (52.9) | 48031 (52.1) | 46472 (50.4) | 42733 (46.4) |
| £52,000~£100,000 | 21389 (23.2) | 18359 (19.9) | 16859 (18.3) | 16296 (17.7) | 15040 (16.3) |
| >£100,000 | 6362 (6.9) | 4337 (4.7) | 3555 (3.9) | 3799 (4.1) | 5179 (5.6) |
| Smoke, n (%) |  |  |  |  |  |
| Never | 53418 (58) | 52007 (56.4) | 50760 (55.1) | 49659 (53.9) | 46580 (50.5) |
| Previous | 31956 (34.7) | 32267 (35) | 32088 (34.8) | 31825 (34.5) | 32245 (35) |
| Current | 6801 (7.4) | 7900 (8.6) | 9327 (10.1) | 10690 (11.6) | 13349 (14.5) |
| Drink, n (%) |  |  |  |  |  |
| Never | 2737 (3) | 3510 (3.8) | 4215 (4.6) | 4957 (5.4) | 6048 (6.6) |
| Previous | 2524 (2.7) | 2878 (3.1) | 3278 (3.6) | 3548 (3.8) | 4285 (4.6) |
| Current | 86914 (94.3) | 85786 (93.1) | 84682 (91.9) | 83669 (90.8) | 81841 (88.8) |
| Hypertension, n (%) | 23728 (25.7) | 25210 (27.4) | 25650 (27.8) | 25800 (28) | 25727 (27.9) |
| Diabetes, n (%) | 3697 (4) | 4483 (4.9) | 4785 (5.2) | 5194 (5.6) | 5659 (6.1) |
| CVD, n (%) | 12806 (13.9) | 13910 (15.1) | 14205 (15.4) | 14432 (15.7) | 14630 (15.9) |
| CAD, n (%) | 10449 (11.3) | 11520 (12.5) | 11740 (12.7) | 11979 (13) | 12036 (13.1) |
| Stroke, n (%) | 3279 (3.6) | 3543 (3.8) | 3643 (4) | 3633 (3.9) | 3926 (4.3) |

**Supplementary Table S5. Pearson correlation coefficients among the five air pollutants and four cognitive impairment subtypes in the UK Biobank**

|  | **NO_2_** | **NO** | **PM_10_** | **PM_2.5_** | **PM_2.5-10_** | Air pollution score for **All-cause dementia** | Air pollution score for **Alzheimer’s dementia** | Air pollution score for **Vascular dementia** | Air pollution score for **MCI** |
| --- | --- | --- | --- | --- | --- | --- | --- | --- | --- |
| **NO_2_** | 1 | - | - | - | - | - | - | - | - |
| **NO** | 0.92211^*^ | 1 | - | - | - | - | - | - | - |
| **PM_10_** | 0.5056^*^ | 0.51246^*^ | 1 | - | - | - | - | - | - |
| **PM_2.5_** | 0.86448^*^ | 0.84718^*^ | 0.53266^*^ | 1 | - | - | - | - | - |
| **PM_2.5-10_** | 0.20205^*^ | 0.23986^*^ | 0.81797^*^ | 0.22255^*^ | 1 | - | - | - | - |
| Air pollution score for **all-cause dementia** | 0.96015^*^ | 0.94738^*^ | 0.65229^*^ | 0.93853^*^ | 0.3429^*^ | 1 | - | - | - |
| Air pollution score for **Alzheimer’s dementia** | 0.95703^*^ | 0.94919^*^ | 0.65481^*^ | 0.93905^*^ | 0.34621^*^ | 0.99991^*^ | 1 | - | - |
| Air pollution score for **vascular dementia** | 0.92386^*^ | 0.91259^*^ | 0.7688^*^ | 0.90267^*^ | 0.46854^*^ | 0.98602^*^ | 0.98638^*^ | 1 | - |
| Air pollution score for **MCI** | 0.92591^*^ | 0.92218^*^ | 0.75222^*^ | 0.9069^*^ | 0.48252^*^ | 0.98778^*^ | 0.98832^*^ | 0.99774^*^ | 1 |
| PM_2.5_: particular matter with an aerodynamic diameter≤2.5 µm; PM_10_: particular matter with an aerodynamic diameter≤10 µm; PM_2.5-10_: particular matter with an aerodynamic diameter between 2.5 and 10 µm; NO_2_: nitrogen dioxide; NO: nitrogen oxides.  * P<0.001 | | | | | | | | | |

**Supplementary Table S6. The HRs and 95% CIs for a 10 µg/m^3^ increase in single air pollutant concentrations with risk of cognitive impairments, adjusted by potential confounders**

|  | Q1 (39.22, 49.82) | Q2 (49.82, 54.61) | Q3 (49.82, 54.61) | Q4 (58.38, 63.09) | Q5 (63.09, 157.77) | P for trend | HR per 10 |
| --- | --- | --- | --- | --- | --- | --- | --- |
| **All-cause dementia** |  |  |  |  |  |  |  |
| NO_2_ |  |  |  |  |  |  |  |
| Daily intake | 17.16 (12.93, 20.19) | 22.42 (20.2, 24.32) | 26.15 (24.33, 28.12) | 30.12 (28.13, 32.5) | 36.07 (32.51, 108.49) |  |  |
| Case/person-years | 1253/1156119 | 1383/1151756 | 1487/1148429 | 1451/1144441 | 1422/1138067 |  |  |
| Model1 | Reference | 1.12 (1.04 - 1.21) | 1.25 (1.16 - 1.35) | 1.29 (1.2 - 1.39) | 1.43 (1.33 - 1.55) | 0.00000000 | 1.17 (1.14 - 1.21) |
| Model2 | Reference | 1.05 (0.97 - 1.14) | 1.14 (1.05 - 1.22) | 1.16 (1.07 - 1.25) | 1.26 (1.16 - 1.36) | 0.00000000 | 1.11 (1.08 - 1.15) |
| Model3 | Reference | 1.03 (0.96 - 1.11) | 1.11 (1.03 - 1.2) | 1.13 (1.04 - 1.22) | 1.21 (1.12 - 1.31) | 0.00000013 | 1.1 (1.06 - 1.13) |
| NO |  |  |  |  |  |  |  |
| Daily intake | 26.51 (19.74, 32.14) | 36.06 (32.15, 39.24) | 42.25 (39.25, 45.28) | 48.7 (45.29, 53.25) | 61.65 (53.26, 265.94) |  |  |
| Case/person-years | 1251/1155842 | 1397/1148942 | 1414/1144103 | 1496/1143671 | 1438/1146253 |  |  |
| Model1 | Reference | 1.15 (1.07 - 1.24) | 1.2 (1.11 - 1.3) | 1.35 (1.25 - 1.46) | 1.42 (1.31 - 1.53) | 0.00000000 | 1.07 (1.05 - 1.08) |
| Model2 | Reference | 1.09 (1.01 - 1.17) | 1.1 (1.01 - 1.18) | 1.2 (1.11 - 1.3) | 1.23 (1.14 - 1.33) | 0.00000001 | 1.04 (1.02 - 1.05) |
| Model3 | Reference | 1.07 (0.99 - 1.16) | 1.07 (0.99 - 1.16) | 1.17 (1.08 - 1.26) | 1.18 (1.09 - 1.28) | 0.00000214 | 1.03 (1.02 - 1.05) |
| PM_10_ |  |  |  |  |  |  |  |
| Daily intake | 14.16 (11.78, 14.96) | 15.5 (14.97, 15.77) | 16.04 (15.78, 16.33) | 16.73 (16.34, 17.36) | 18.71 (17.37, 31.39) |  |  |
| Case/person-years | 1287/1159028 | 1419/1166037 | 1469/1138952 | 1432/1131758 | 1389/1143036 |  |  |
| Model1 | Reference | 1.11 (1.03 - 1.2) | 1.21 (1.13 - 1.31) | 1.23 (1.14 - 1.33) | 1.18 (1.1 - 1.28) | 0.00000028 | 1.37 (1.21 - 1.55) |
| Model2 | Reference | 1.06 (0.98 - 1.14) | 1.14 (1.06 - 1.23) | 1.15 (1.07 - 1.24) | 1.11 (1.02 - 1.19) | 0.00113162 | 1.21 (1.07 - 1.37) |
| Model3 | Reference | 1.05 (0.97 - 1.13) | 1.13 (1.05 - 1.22) | 1.14 (1.05 - 1.23) | 1.09 (1.01 - 1.18) | 0.00286519 | 1.19 (1.05 - 1.34) |
| PM_2.5_ |  |  |  |  |  |  |  |
| Daily intake | 8.66 (8.17, 9.12) | 9.44 (9.13, 9.7) | 9.94 (9.71, 10.16) | 10.42 (10.17, 10.75) | 11.31 (10.76, 21.31) |  |  |
| Case/person-years | 1238/1161075 | 1386/1143725 | 1414/1144666 | 1502/1144338 | 1456/1145007 |  |  |
| Model1 | Reference | 1.17 (1.08 - 1.26) | 1.23 (1.14 - 1.33) | 1.36 (1.26 - 1.47) | 1.42 (1.32 - 1.53) | 0.00000000 | 3.18 (2.57 - 3.94) |
| Model2 | Reference | 1.11 (1.02 - 1.19) | 1.14 (1.05 - 1.23) | 1.21 (1.13 - 1.31) | 1.23 (1.14 - 1.33) | 0.00000001 | 2.02 (1.62 - 2.53) |
| Model3 | Reference | 1.09 (1.01 - 1.18) | 1.12 (1.04 - 1.21) | 1.18 (1.1 - 1.28) | 1.18 (1.09 - 1.28) | 0.00000220 | 1.78 (1.42 - 2.22) |
| PM_2.5-10_ |  |  |  |  |  |  |  |
| Daily intake | 5.7 (5.57, 5.8) | 5.89 (5.81, 5.99) | 6.12 (6, 6.27) | 6.51 (6.28, 6.83) | 7.64 (6.84, 12.82) |  |  |
| Case/person-years | 1415/1203435 | 1394/1126453 | 1426/1152827 | 1361/1109233 | 1400/1146864 |  |  |
| Model1 | Reference | 1.07 (1 - 1.15) | 1.1 (1.02 - 1.18) | 1.11 (1.03 - 1.2) | 1.1 (1.02 - 1.18) | 0.00708323 | 1.23 (0.96 - 1.59) |
| Model2 | Reference | 1.04 (0.97 - 1.12) | 1.05 (0.98 - 1.13) | 1.08 (1 - 1.16) | 1.05 (0.97 - 1.13) | 0.12550757 | 1.12 (0.87 - 1.45) |
| Model3 | Reference | 1.04 (0.96 - 1.12) | 1.05 (0.97 - 1.13) | 1.07 (0.99 - 1.15) | 1.04 (0.97 - 1.12) | 0.20305034 | 1.1 (0.85 - 1.42) |
| **Alzheimer’s dementia** |  |  |  |  |  |  |  |
| NO_2_ |  |  |  |  |  |  |  |
| Daily intake | 17.16 (12.93, 20.19) | 22.42 (20.2, 24.32) | 26.15 (24.33, 28.12) | 30.12 (28.13, 32.5) | 36.07 (32.51, 108.49) |  |  |
| Case/person-years | 541/1157756 | 592/1153659 | 623/1150425 | 567/1146690 | 604/1140213 |  |  |
| Model1 | Reference | 1.11 (0.99 - 1.25) | 1.21 (1.08 - 1.36) | 1.17 (1.04 - 1.31) | 1.42 (1.26 - 1.59) | 0.00000002 | 1.16 (1.11 - 1.21) |
| Model2 | Reference | 1.05 (0.93 - 1.17) | 1.11 (0.99 - 1.24) | 1.06 (0.94 - 1.2) | 1.28 (1.14 - 1.44) | 0.00019208 | 1.12 (1.06 - 1.17) |
| Model3 | Reference | 1.04 (0.93 - 1.17) | 1.1 (0.98 - 1.24) | 1.05 (0.94 - 1.19) | 1.26 (1.12 - 1.42) | 0.00053081 | 1.11 (1.06 - 1.16) |
| NO |  |  |  |  |  |  |  |
| Daily intake | 26.51 (19.74, 32.14) | 36.06 (32.15, 39.24) | 42.25 (39.25, 45.28) | 48.7 (45.29, 53.25) | 61.65 (53.26, 265.94) |  |  |
| Case/person-years | 532/1157500 | 586/1150893 | 588/1146144 | 625/1145764 | 596/1148442 |  |  |
| Model1 | Reference | 1.13 (1.01 - 1.27) | 1.17 (1.04 - 1.32) | 1.33 (1.18 - 1.49) | 1.39 (1.23 - 1.56) | 0.00000000 | 1.07 (1.05 - 1.09) |
| Model2 | Reference | 1.08 (0.96 - 1.21) | 1.08 (0.96 - 1.22) | 1.2 (1.07 - 1.35) | 1.24 (1.1 - 1.39) | 0.00008895 | 1.05 (1.02 - 1.07) |
| Model3 | Reference | 1.08 (0.96 - 1.21) | 1.07 (0.95 - 1.21) | 1.19 (1.06 - 1.34) | 1.22 (1.08 - 1.37) | 0.00025435 | 1.04 (1.02 - 1.07) |
| PM_10_ |  |  |  |  |  |  |  |
| Daily intake | 14.16 (11.78, 14.96) | 15.5 (14.97, 15.77) | 16.04 (15.78, 16.33) | 16.73 (16.34, 17.36) | 18.71 (17.37, 31.39) |  |  |
| Case/person-years | 540/1160843 | 598/1167997 | 624/1140952 | 580/1133852 | 585/1145099 |  |  |
| Model1 | Reference | 1.11 (0.99 - 1.25) | 1.23 (1.09 - 1.38) | 1.19 (1.06 - 1.34) | 1.19 (1.06 - 1.34) | 0.00179107 | 1.37 (1.14 - 1.66) |
| Model2 | Reference | 1.07 (0.95 - 1.2) | 1.16 (1.03 - 1.3) | 1.12 (1 - 1.26) | 1.13 (1 - 1.27) | 0.02978110 | 1.24 (1.03 - 1.51) |
| Model3 | Reference | 1.06 (0.95 - 1.19) | 1.16 (1.03 - 1.3) | 1.12 (1 - 1.26) | 1.13 (1 - 1.27) | 0.02859886 | 1.24 (1.03 - 1.5) |
| PM_2.5_ |  |  |  |  |  |  |  |
| Daily intake | 8.66 (8.17, 9.12) | 9.44 (9.13, 9.7) | 9.94 (9.71, 10.16) | 10.42 (10.17, 10.75) | 11.31 (10.76, 21.31) |  |  |
| Case/person-years | 534/1162791 | 591/1145599 | 566/1146649 | 613/1146488 | 623/1147216 |  |  |
| Model1 | Reference | 1.15 (1.03 - 1.3) | 1.14 (1.01 - 1.28) | 1.29 (1.15 - 1.45) | 1.42 (1.26 - 1.59) | 0.00000000 | 3.15 (2.27 - 4.39) |
| Model2 | Reference | 1.1 (0.98 - 1.23) | 1.07 (0.95 - 1.2) | 1.17 (1.04 - 1.31) | 1.26 (1.12 - 1.42) | 0.00010595 | 2.19 (1.55 - 3.08) |
| Model3 | Reference | 1.09 (0.97 - 1.23) | 1.06 (0.94 - 1.2) | 1.16 (1.03 - 1.3) | 1.24 (1.1 - 1.39) | 0.00035157 | 2.08 (1.47 - 2.93) |
| PM_2.5-10_ |  |  |  |  |  |  |  |
| Daily intake | 5.7 (5.57, 5.8) | 5.89 (5.81, 5.99) | 6.12 (6, 6.27) | 6.51 (6.28, 6.83) | 7.64 (6.84, 12.82) |  |  |
| Case/person-years | 600/1205316 | 579/1128424 | 601/1154914 | 560/1111191 | 587/1148898 |  |  |
| Model1 | Reference | 1.05 (0.94 - 1.18) | 1.09 (0.97 - 1.22) | 1.08 (0.97 - 1.22) | 1.09 (0.97 - 1.22) | 0.11728326 | 1.24 (0.83 - 1.84) |
| Model2 | Reference | 1.02 (0.91 - 1.14) | 1.05 (0.94 - 1.18) | 1.05 (0.94 - 1.18) | 1.05 (0.94 - 1.18) | 0.31511969 | 1.15 (0.77 - 1.71) |
| Model3 | Reference | 1.02 (0.91 - 1.14) | 1.05 (0.94 - 1.18) | 1.06 (0.94 - 1.18) | 1.05 (0.94 - 1.18) | 0.31209224 | 1.15 (0.77 - 1.71) |
| **Vascular dementia** |  |  |  |  |  |  |  |
| NO_2_ |  |  |  |  |  |  |  |
| Daily intake | 17.16 (12.93, 20.19) | 22.42 (20.2, 24.32) | 26.15 (24.33, 28.12) | 30.12 (28.13, 32.5) | 36.07 (32.51, 108.49) |  |  |
| Case/person-years | 248/1158431 | 301/1154404 | 351/1151167 | 331/1147340 | 313/1141049 |  |  |
| Model1 | Reference | 1.23 (1.04 - 1.46) | 1.49 (1.27 - 1.76) | 1.49 (1.27 - 1.76) | 1.61 (1.37 - 1.91) | 0.00000000 | 1.21 (1.14 - 1.29) |
| Model2 | Reference | 1.13 (0.95 - 1.34) | 1.31 (1.11 - 1.54) | 1.29 (1.09 - 1.52) | 1.35 (1.14 - 1.6) | 0.00016014 | 1.13 (1.06 - 1.21) |
| Model3 | Reference | 1.08 (0.91 - 1.28) | 1.24 (1.06 - 1.47) | 1.2 (1.02 - 1.42) | 1.23 (1.04 - 1.46) | 0.00746658 | 1.09 (1.02 - 1.16) |
| NO |  |  |  |  |  |  |  |
| Daily intake | 26.51 (19.74, 32.14) | 36.06 (32.15, 39.24) | 42.25 (39.25, 45.28) | 48.7 (45.29, 53.25) | 61.65 (53.26, 265.94) |  |  |
| Case/person-years | 250/1158160 | 288/1151686 | 357/1146655 | 322/1146677 | 327/1149213 |  |  |
| Model1 | Reference | 1.19 (1 - 1.4) | 1.52 (1.3 - 1.79) | 1.46 (1.24 - 1.73) | 1.63 (1.38 - 1.92) | 0.00000000 | 1.08 (1.05 - 1.11) |
| Model2 | Reference | 1.1 (0.93 - 1.3) | 1.34 (1.14 - 1.58) | 1.25 (1.05 - 1.47) | 1.34 (1.13 - 1.59) | 0.00023905 | 1.04 (1.01 - 1.08) |
| Model3 | Reference | 1.07 (0.9 - 1.26) | 1.27 (1.08 - 1.5) | 1.16 (0.98 - 1.38) | 1.22 (1.03 - 1.45) | 0.01261393 | 1.03 (0.99 - 1.06) |
| PM_10_ |  |  |  |  |  |  |  |
| Daily intake | 14.16 (11.78, 14.96) | 15.5 (14.97, 15.77) | 16.04 (15.78, 16.33) | 16.73 (16.34, 17.36) | 18.71 (17.37, 31.39) |  |  |
| Case/person-years | 268/1161547 | 327/1168792 | 325/1141727 | 292/1134497 | 332/1145827 |  |  |
| Model1 | Reference | 1.23 (1.05 - 1.45) | 1.29 (1.1 - 1.52) | 1.21 (1.03 - 1.43) | 1.37 (1.16 - 1.61) | 0.00088959 | 1.77 (1.37 - 2.28) |
| Model2 | Reference | 1.15 (0.98 - 1.36) | 1.19 (1.01 - 1.39) | 1.11 (0.94 - 1.31) | 1.24 (1.06 - 1.46) | 0.03409408 | 1.52 (1.17 - 1.97) |
| Model3 | Reference | 1.12 (0.96 - 1.32) | 1.16 (0.99 - 1.36) | 1.08 (0.91 - 1.27) | 1.21 (1.03 - 1.42) | 0.07687753 | 1.44 (1.11 - 1.87) |
| PM_2.5_ |  |  |  |  |  |  |  |
| Daily intake | 8.66 (8.17, 9.12) | 9.44 (9.13, 9.7) | 9.94 (9.71, 10.16) | 10.42 (10.17, 10.75) | 11.31 (10.76, 21.31) |  |  |
| Case/person-years | 248/1163455 | 321/1146346 | 308/1147260 | 348/1147244 | 319/1148086 |  |  |
| Model1 | Reference | 1.35 (1.14 - 1.59) | 1.34 (1.13 - 1.59) | 1.58 (1.34 - 1.86) | 1.57 (1.33 - 1.85) | 0.00000001 | 3.82 (2.44 - 5.99) |
| Model2 | Reference | 1.26 (1.06 - 1.48) | 1.21 (1.02 - 1.43) | 1.35 (1.15 - 1.6) | 1.29 (1.09 - 1.52) | 0.00300669 | 2.05 (1.28 - 3.28) |
| Model3 | Reference | 1.21 (1.02 - 1.43) | 1.16 (0.98 - 1.38) | 1.27 (1.07 - 1.49) | 1.17 (0.99 - 1.38) | 0.07946402 | 1.52 (0.94 - 2.44) |
| PM_2.5-10_ |  |  |  |  |  |  |  |
| Daily intake | 5.7 (5.57, 5.8) | 5.89 (5.81, 5.99) | 6.12 (6, 6.27) | 6.51 (6.28, 6.83) | 7.64 (6.84, 12.82) |  |  |
| Case/person-years | 303/1206191 | 309/1129025 | 318/1155657 | 280/1111918 | 334/1149600 |  |  |
| Model1 | Reference | 1.11 (0.95 - 1.3) | 1.14 (0.98 - 1.34) | 1.08 (0.92 - 1.27) | 1.23 (1.05 - 1.43) | 0.02896432 | 1.9 (1.13 - 3.21) |
| Model2 | Reference | 1.07 (0.91 - 1.25) | 1.08 (0.92 - 1.27) | 1.03 (0.87 - 1.21) | 1.15 (0.99 - 1.35) | 0.15266093 | 1.68 (0.99 - 2.86) |
| Model3 | Reference | 1.06 (0.9 - 1.24) | 1.06 (0.91 - 1.24) | 1 (0.85 - 1.18) | 1.13 (0.96 - 1.32) | 0.27547464 | 1.6 (0.94 - 2.73) |
| **MCI** |  |  |  |  |  |  |  |
| NO_2_ |  |  |  |  |  |  |  |
| Daily intake | 17.16 (12.93, 20.19) | 22.42 (20.2, 24.32) | 26.15 (24.33, 28.12) | 30.12 (28.13, 32.5) | 36.07 (32.51, 108.49) |  |  |
| Case/person-years | 131/1158587 | 153/1154616 | 154/1151497 | 187/1147478 | 219/1141025 |  |  |
| Model1 | Reference | 1.19 (0.94 - 1.5) | 1.23 (0.98 - 1.55) | 1.57 (1.25 - 1.96) | 2.03 (1.63 - 2.52) | 0.00000000 | 1.37 (1.27 - 1.48) |
| Model2 | Reference | 1.11 (0.88 - 1.41) | 1.12 (0.88 - 1.41) | 1.38 (1.1 - 1.73) | 1.7 (1.36 - 2.12) | 0.00000025 | 1.29 (1.19 - 1.4) |
| Model3 | Reference | 1.08 (0.86 - 1.37) | 1.08 (0.86 - 1.37) | 1.32 (1.06 - 1.66) | 1.61 (1.29 - 2.02) | 0.00000268 | 1.27 (1.17 - 1.38) |
| NO |  |  |  |  |  |  |  |
| Daily intake | 26.51 (19.74, 32.14) | 36.06 (32.15, 39.24) | 42.25 (39.25, 45.28) | 48.7 (45.29, 53.25) | 61.65 (53.26, 265.94) |  |  |
| Case/person-years | 136/1158298 | 140/1151861 | 144/1147114 | 190/1146753 | 234/1149177 |  |  |
| Model1 | Reference | 1.06 (0.83 - 1.34) | 1.12 (0.88 - 1.41) | 1.55 (1.24 - 1.93) | 2.05 (1.66 - 2.53) | 0.00000000 | 1.13 (1.1 - 1.17) |
| Model2 | Reference | 1 (0.79 - 1.26) | 1.01 (0.8 - 1.28) | 1.36 (1.09 - 1.7) | 1.71 (1.37 - 2.12) | 0.00000001 | 1.11 (1.07 - 1.14) |
| Model3 | Reference | 0.98 (0.77 - 1.24) | 0.98 (0.77 - 1.24) | 1.3 (1.04 - 1.63) | 1.62 (1.3 - 2.01) | 0.00000014 | 1.1 (1.06 - 1.14) |
| PM_10_ |  |  |  |  |  |  |  |
| Daily intake | 14.16 (11.78, 14.96) | 15.5 (14.97, 15.77) | 16.04 (15.78, 16.33) | 16.73 (16.34, 17.36) | 18.71 (17.37, 31.39) |  |  |
| Case/person-years | 128/1161809 | 159/1168984 | 167/1141944 | 181/1134579 | 209/1145887 |  |  |
| Model1 | Reference | 1.25 (0.99 - 1.58) | 1.38 (1.1 - 1.74) | 1.55 (1.23 - 1.94) | 1.77 (1.42 - 2.2) | 0.00000005 | 2.31 (1.65 - 3.23) |
| Model2 | Reference | 1.18 (0.94 - 1.49) | 1.27 (1.01 - 1.6) | 1.41 (1.12 - 1.77) | 1.6 (1.28 - 1.99) | 0.00000871 | 1.97 (1.4 - 2.78) |
| Model3 | Reference | 1.17 (0.92 - 1.47) | 1.26 (1 - 1.59) | 1.39 (1.1 - 1.74) | 1.57 (1.26 - 1.96) | 0.00001736 | 1.91 (1.35 - 2.69) |
| PM_2.5_ |  |  |  |  |  |  |  |
| Daily intake | 8.66 (8.17, 9.12) | 9.44 (9.13, 9.7) | 9.94 (9.71, 10.16) | 10.42 (10.17, 10.75) | 11.31 (10.76, 21.31) |  |  |
| Case/person-years | 133/1163668 | 138/1146575 | 162/1147522 | 188/1147418 | 223/1148020 |  |  |
| Model1 | Reference | 1.08 (0.85 - 1.37) | 1.3 (1.03 - 1.63) | 1.56 (1.25 - 1.94) | 1.97 (1.59 - 2.44) | 0.00000000 | 8.43 (4.75 - 14.98) |
| Model2 | Reference | 1.02 (0.8 - 1.29) | 1.19 (0.95 - 1.5) | 1.37 (1.09 - 1.71) | 1.64 (1.31 - 2.04) | 0.00000018 | 4.83 (2.64 - 8.83) |
| Model3 | Reference | 0.99 (0.78 - 1.26) | 1.16 (0.92 - 1.47) | 1.32 (1.06 - 1.65) | 1.55 (1.24 - 1.93) | 0.00000253 | 4.06 (2.21 - 7.47) |
| PM_2.5-10_ |  |  |  |  |  |  |  |
| Daily intake | 5.7 (5.57, 5.8) | 5.89 (5.81, 5.99) | 6.12 (6, 6.27) | 6.51 (6.28, 6.83) | 7.64 (6.84, 12.82) |  |  |
| Case/person-years | 159/1206382 | 138/1129332 | 176/1155792 | 178/1112017 | 193/1149681 |  |  |
| Model1 | Reference | 0.94 (0.75 - 1.18) | 1.2 (0.97 - 1.48) | 1.28 (1.03 - 1.59) | 1.33 (1.08 - 1.64) | 0.00034289 | 2.46 (1.23 - 4.92) |
| Model2 | Reference | 0.91 (0.72 - 1.14) | 1.13 (0.92 - 1.41) | 1.21 (0.98 - 1.5) | 1.24 (1.01 - 1.53) | 0.00373010 | 2.14 (1.06 - 4.31) |
| Model3 | Reference | 0.9 (0.72 - 1.13) | 1.12 (0.91 - 1.39) | 1.19 (0.96 - 1.48) | 1.22 (0.99 - 1.51) | 0.00594679 | 2.06 (1.02 - 4.17) |
| Model1 was adjusted for age and sex.  Model2 was adjusted for age, sex, ethnicity, income, employment, education, drink, and smoke.  Model3 was adjusted for age, sex, ethnicity, income, employment, education, drink, smoke, MET, BMI, SBP, hypertension, CVD, and diabetes. | | | | | | | |


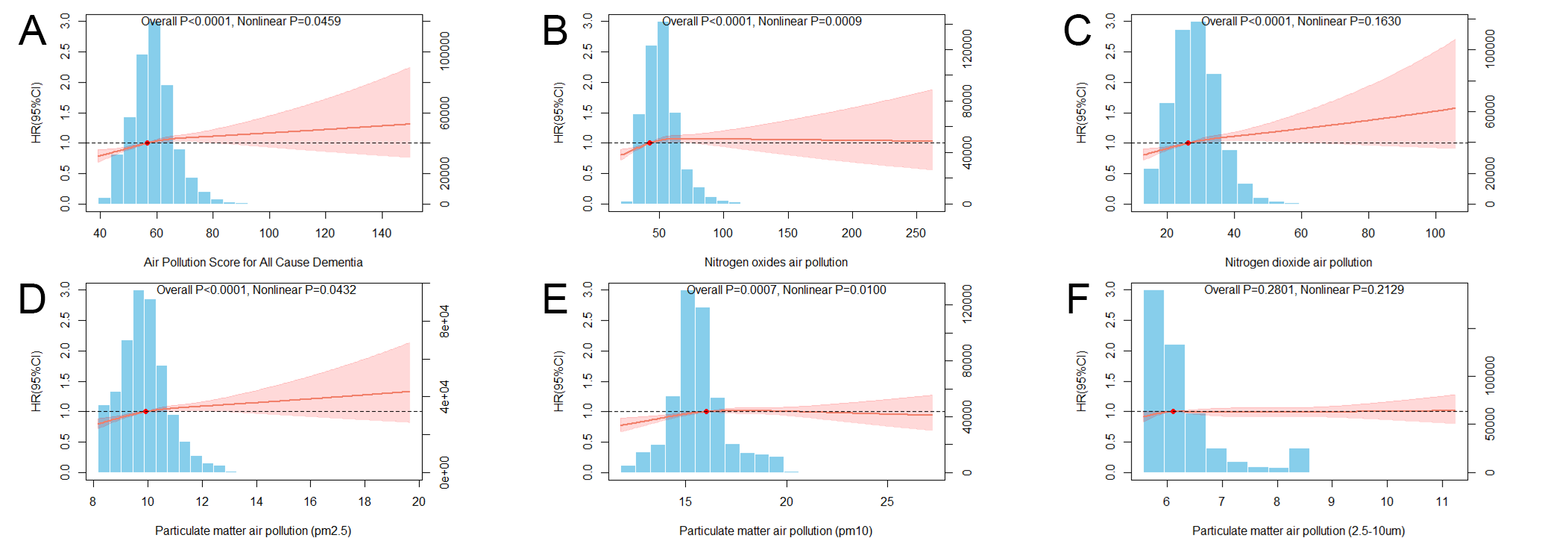


**Supplementary Figure S2. All-cause dementia. (A) Air pollution score. (B) NO. (C) NO_2_. (D) PM_2.5_. (E) PM_10_. (F) PM_2.5-10_.**


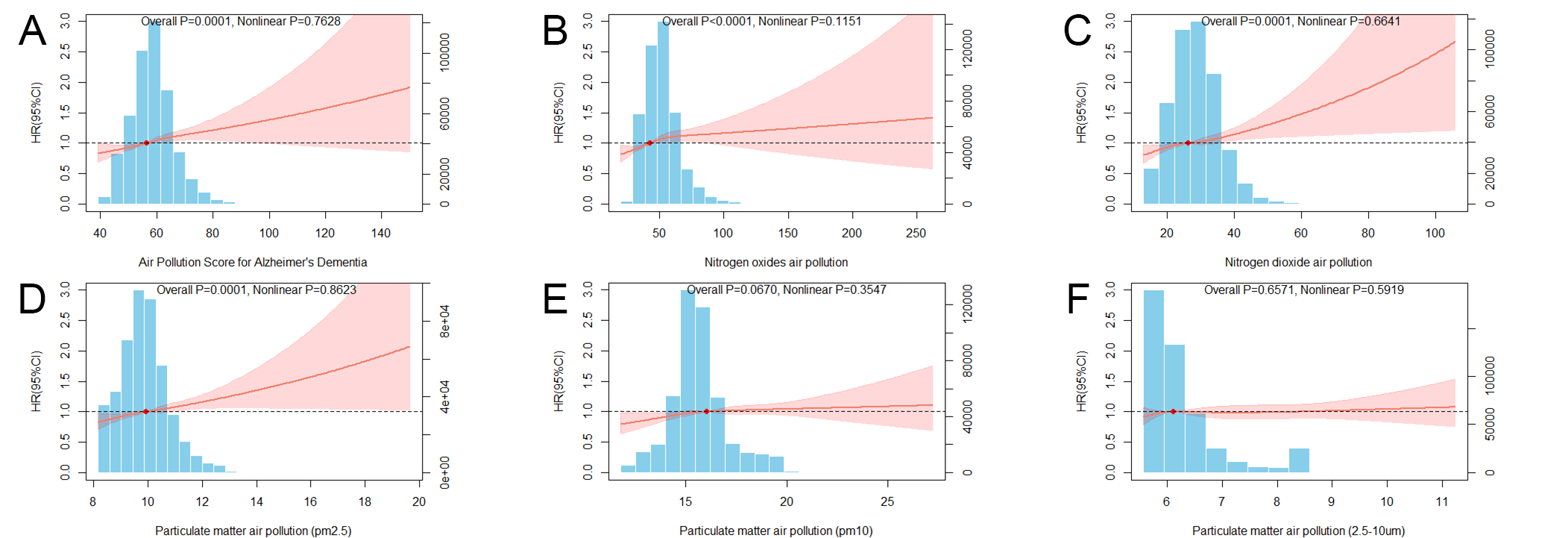


**Supplementary Figure S3. Alzheimer's dementia. (A) Air pollution score. (B) NO. (C) NO_2_. (D) PM_2.5_. (E) PM_10_. (F) PM_2.5-10_.**


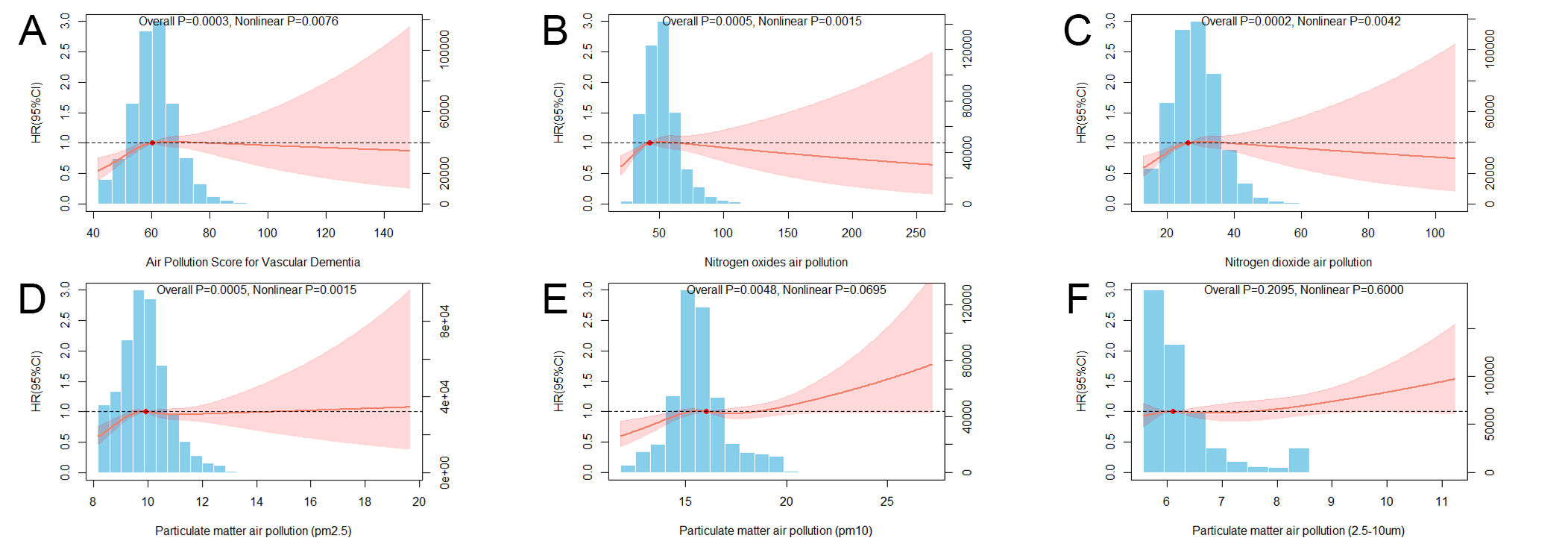


**Supplementary Figure S4. Vascular dementia. (A) Air pollution score. (B) NO. (C) NO_2_. (D) PM_2.5_. (E) PM_10_. (F) PM_2.5-10_.**


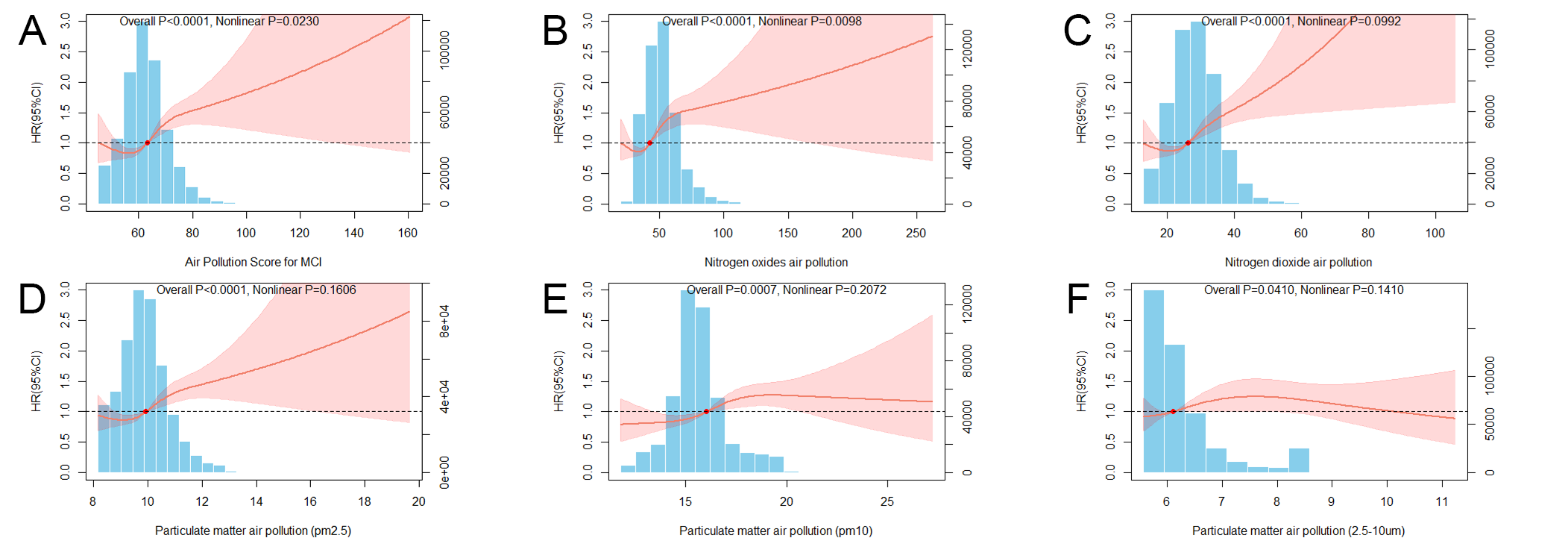


**Supplementary Figure S5. MCI. (A) Air pollution score. (B) NO. (C) NO_2_. (D) PM_2.5_. (E) PM_10_. (F) PM_2.5-10_.**

**Supplementary Table S7. The analyses stratified by the genetic predisposition according to the APOE gene status**

| Outcome | Exposure | Subgroup | Q1 | Q2 | Q3 | Q4 | Q5 | P for interaction |
| --- | --- | --- | --- | --- | --- | --- | --- | --- |
| All-cause dementia | Air pollution score |  |  |  |  |  |  |  |
|  |  | APOE |  |  |  |  |  |  |
|  |  | 0 | Ref | 1.15 (1.01 - 1.3) | 1.15 (1.01 - 1.3) | 1.26 (1.11 - 1.42) | 1.31 (1.15 - 1.49) | 0.245316235 |
|  |  | 1 | Ref | 0.98 (0.86 - 1.12) | 1.14 (1 - 1.29) | 1.13 (0.99 - 1.29) | 1.16 (1.01 - 1.33) |  |
|  |  | 2 | Ref | 1.07 (0.83 - 1.37) | 1.01 (0.79 - 1.3) | 1.1 (0.85 - 1.42) | 1.35 (1.04 - 1.75) |  |
| All-cause dementia | NO_2_ |  |  |  |  |  |  |  |
|  |  | APOE |  |  |  |  |  |  |
|  |  | 0 | Ref | 1.09 (0.96 - 1.23) | 1.11 (0.98 - 1.25) | 1.14 (1.01 - 1.29) | 1.25 (1.11 - 1.42) | 0.317320992 |
|  |  | 1 | Ref | 0.98 (0.86 - 1.12) | 1.13 (0.99 - 1.28) | 1.17 (1.03 - 1.33) | 1.19 (1.04 - 1.37) |  |
|  |  | 2 | Ref | 1.07 (0.83 - 1.37) | 1.09 (0.85 - 1.4) | 1.01 (0.77 - 1.31) | 1.47 (1.14 - 1.89) |  |
| All-cause dementia | NO |  |  |  |  |  |  |  |
|  |  | APOE |  |  |  |  |  |  |
|  |  | 0 | Ref | 1.17 (1.04 - 1.33) | 1.11 (0.98 - 1.26) | 1.2 (1.06 - 1.36) | 1.28 (1.13 - 1.45) | 0.06691002 |
|  |  | 1 | Ref | 1.04 (0.91 - 1.18) | 0.99 (0.87 - 1.13) | 1.21 (1.07 - 1.38) | 1.16 (1.01 - 1.32) |  |
|  |  | 2 | Ref | 0.94 (0.73 - 1.2) | 1.09 (0.85 - 1.39) | 0.9 (0.7 - 1.16) | 1.27 (0.98 - 1.63) |  |
| All-cause dementia | PM_10_ |  |  |  |  |  |  |  |
|  |  | APOE |  |  |  |  |  |  |
|  |  | 0 | Ref | 1.12 (0.99 - 1.27) | 1.23 (1.08 - 1.39) | 1.25 (1.11 - 1.42) | 1.21 (1.07 - 1.37) | 0.531972334 |
|  |  | 1 | Ref | 1.03 (0.91 - 1.17) | 1.09 (0.96 - 1.24) | 1.09 (0.95 - 1.24) | 1.03 (0.9 - 1.18) |  |
|  |  | 2 | Ref | 0.95 (0.74 - 1.22) | 1.09 (0.85 - 1.39) | 1.12 (0.87 - 1.43) | 1.05 (0.81 - 1.36) |  |
| All-cause dementia | PM_2.5_ |  |  |  |  |  |  |  |
|  |  | APOE |  |  |  |  |  |  |
|  |  | 0 | Ref | 1.18 (1.04 - 1.34) | 1.19 (1.05 - 1.35) | 1.27 (1.12 - 1.44) | 1.33 (1.17 - 1.51) | 0.226016454 |
|  |  | 1 | Ref | 1.01 (0.89 - 1.15) | 1.09 (0.96 - 1.24) | 1.1 (0.96 - 1.25) | 1.1 (0.97 - 1.26) |  |
|  |  | 2 | Ref | 1 (0.78 - 1.29) | 1.1 (0.85 - 1.42) | 1.3 (1.02 - 1.67) | 1.24 (0.95 - 1.61) |  |
| All-cause dementia | PM_2.5-10_ |  |  |  |  |  |  |  |
|  |  | APOE |  |  |  |  |  |  |
|  |  | 0 | Ref | 1.09 (0.97 - 1.24) | 1.2 (1.06 - 1.35) | 1.15 (1.01 - 1.3) | 1.18 (1.05 - 1.34) | 0.104153752 |
|  |  | 1 | Ref | 0.99 (0.87 - 1.12) | 1.01 (0.89 - 1.14) | 1.01 (0.89 - 1.14) | 0.98 (0.87 - 1.12) |  |
|  |  | 2 | Ref | 1.17 (0.92 - 1.49) | 0.9 (0.69 - 1.17) | 1.05 (0.81 - 1.35) | 1.14 (0.88 - 1.46) |  |
| Alzheimer’s dementia | Air pollution score |  |  |  |  |  |  |  |
|  |  | APOE |  |  |  |  |  |  |
|  |  | 0 | Ref | 1.09 (0.88 - 1.35) | 0.92 (0.74 - 1.15) | 1.2 (0.97 - 1.49) | 1.32 (1.07 - 1.63) | 0.142980272 |
|  |  | 1 | Ref | 1.06 (0.87 - 1.28) | 1.2 (1 - 1.45) | 1.15 (0.95 - 1.39) | 1.24 (1.02 - 1.51) |  |
|  |  | 2 | Ref | 1.11 (0.8 - 1.54) | 0.88 (0.62 - 1.24) | 1.04 (0.74 - 1.47) | 1.4 (1 - 1.97) |  |
| Alzheimer’s dementia | NO_2_ |  |  |  |  |  |  |  |
|  |  | APOE |  |  |  |  |  |  |
|  |  | 0 | Ref | 0.93 (0.76 - 1.16) | 1 (0.81 - 1.24) | 1.02 (0.82 - 1.26) | 1.2 (0.97 - 1.49) | 0.369788955 |
|  |  | 1 | Ref | 0.97 (0.81 - 1.18) | 1.14 (0.95 - 1.37) | 1.1 (0.91 - 1.33) | 1.25 (1.02 - 1.51) |  |
|  |  | 2 | Ref | 1.15 (0.83 - 1.58) | 1.02 (0.73 - 1.42) | 0.81 (0.56 - 1.17) | 1.47 (1.05 - 2.06) |  |
| Alzheimer’s dementia | NO |  |  |  |  |  |  |  |
|  |  | APOE |  |  |  |  |  |  |
|  |  | 0 | Ref | 0.98 (0.79 - 1.21) | 0.95 (0.76 - 1.18) | 1.14 (0.92 - 1.4) | 1.2 (0.97 - 1.49) | 0.653713839 |
|  |  | 1 | Ref | 1.12 (0.93 - 1.35) | 1.09 (0.9 - 1.32) | 1.27 (1.05 - 1.53) | 1.22 (1 - 1.48) |  |
|  |  | 2 | Ref | 1.05 (0.75 - 1.46) | 1.08 (0.77 - 1.5) | 0.97 (0.69 - 1.36) | 1.3 (0.92 - 1.83) |  |
| Alzheimer’s dementia | PM_10_ |  |  |  |  |  |  |  |
|  |  | APOE |  |  |  |  |  |  |
|  |  | 0 | Ref | 1.14 (0.92 - 1.4) | 1.17 (0.95 - 1.45) | 1.13 (0.91 - 1.4) | 1.14 (0.92 - 1.41) | 0.955545111 |
|  |  | 1 | Ref | 1.11 (0.91 - 1.34) | 1.27 (1.05 - 1.54) | 1.3 (1.07 - 1.58) | 1.21 (1 - 1.47) |  |
|  |  | 2 | Ref | 0.93 (0.67 - 1.3) | 1.11 (0.8 - 1.54) | 1.11 (0.8 - 1.55) | 1 (0.7 - 1.41) |  |
| Alzheimer’s dementia | PM_2.5_ |  |  |  |  |  |  |  |
|  |  | APOE |  |  |  |  |  |  |
|  |  | 0 | Ref | 1.15 (0.93 - 1.43) | 1.02 (0.81 - 1.27) | 1.19 (0.96 - 1.48) | 1.4 (1.14 - 1.74) | 0.188233386 |
|  |  | 1 | Ref | 1.05 (0.87 - 1.26) | 1.13 (0.94 - 1.36) | 1.03 (0.85 - 1.25) | 1.18 (0.97 - 1.42) |  |
|  |  | 2 | Ref | 0.88 (0.62 - 1.24) | 0.98 (0.7 - 1.39) | 1.26 (0.91 - 1.74) | 1.2 (0.85 - 1.7) |  |
| Alzheimer’s dementia | PM_2.5-10_ |  |  |  |  |  |  |  |
|  |  | APOE |  |  |  |  |  |  |
|  |  | 0 | Ref | 0.99 (0.8 - 1.22) | 1.2 (0.98 - 1.47) | 1.02 (0.83 - 1.27) | 1.09 (0.89 - 1.35) | 0.756326298 |
|  |  | 1 | Ref | 1.04 (0.86 - 1.26) | 1.14 (0.95 - 1.37) | 1.12 (0.93 - 1.35) | 1.12 (0.93 - 1.35) |  |
|  |  | 2 | Ref | 1.05 (0.76 - 1.45) | 0.85 (0.6 - 1.19) | 0.99 (0.71 - 1.38) | 1.02 (0.73 - 1.42) |  |
| Vascular dementia | Air pollution score |  |  |  |  |  |  |  |
|  |  | APOE |  |  |  |  |  |  |
|  |  | 0 | Ref | 1.19 (0.91 - 1.55) | 1.21 (0.93 - 1.57) | 1.07 (0.81 - 1.4) | 1.38 (1.06 - 1.79) | 0.290938317 |
|  |  | 1 | Ref | 1.14 (0.84 - 1.55) | 1.56 (1.17 - 2.09) | 1.43 (1.06 - 1.93) | 1.43 (1.05 - 1.95) |  |
|  |  | 2 | Ref | 1.24 (0.73 - 2.1) | 0.85 (0.48 - 1.52) | 1.2 (0.69 - 2.08) | 1.06 (0.58 - 1.92) |  |
| Vascular dementia | NO_2_ |  |  |  |  |  |  |  |
|  |  | APOE |  |  |  |  |  |  |
|  |  | 0 | Ref | 1.12 (0.86 - 1.45) | 1.06 (0.82 - 1.39) | 1.15 (0.88 - 1.5) | 1.27 (0.97 - 1.65) | 0.384210974 |
|  |  | 1 | Ref | 1.03 (0.75 - 1.4) | 1.55 (1.16 - 2.05) | 1.37 (1.02 - 1.84) | 1.29 (0.94 - 1.77) |  |
|  |  | 2 | Ref | 1.06 (0.6 - 1.86) | 1.07 (0.61 - 1.88) | 1.17 (0.67 - 2.07) | 1.44 (0.81 - 2.55) |  |
| Vascular dementia | NO |  |  |  |  |  |  |  |
|  |  | APOE |  |  |  |  |  |  |
|  |  | 0 | Ref | 1.36 (1.04 - 1.78) | 1.31 (1 - 1.71) | 1.22 (0.93 - 1.61) | 1.41 (1.07 - 1.86) | 0.577335909 |
|  |  | 1 | Ref | 1.05 (0.77 - 1.43) | 1.38 (1.03 - 1.84) | 1.35 (1.01 - 1.81) | 1.36 (1.01 - 1.84) |  |
|  |  | 2 | Ref | 0.85 (0.49 - 1.47) | 0.89 (0.52 - 1.54) | 0.81 (0.47 - 1.41) | 1.14 (0.66 - 1.96) |  |
| Vascular dementia | PM_10_ |  |  |  |  |  |  |  |
|  |  | APOE |  |  |  |  |  |  |
|  |  | 0 | Ref | 1.21 (0.93 - 1.57) | 1.07 (0.82 - 1.4) | 1.26 (0.97 - 1.63) | 1.22 (0.94 - 1.59) | 0.597038763 |
|  |  | 1 | Ref | 1.12 (0.84 - 1.5) | 1.24 (0.93 - 1.65) | 1.12 (0.83 - 1.51) | 1.29 (0.96 - 1.71) |  |
|  |  | 2 | Ref | 1.1 (0.64 - 1.9) | 1.41 (0.83 - 2.39) | 0.88 (0.49 - 1.59) | 1.02 (0.57 - 1.84) |  |
| Vascular dementia | PM_2.5_ |  |  |  |  |  |  |  |
|  |  | APOE |  |  |  |  |  |  |
|  |  | 0 | Ref | 1.32 (1.02 - 1.71) | 1.21 (0.93 - 1.58) | 1.13 (0.87 - 1.48) | 1.2 (0.91 - 1.57) | 0.722848348 |
|  |  | 1 | Ref | 1.26 (0.94 - 1.7) | 1.21 (0.9 - 1.64) | 1.53 (1.14 - 2.03) | 1.25 (0.92 - 1.7) |  |
|  |  | 2 | Ref | 1.3 (0.73 - 2.31) | 1.57 (0.89 - 2.75) | 1.33 (0.75 - 2.37) | 1.29 (0.7 - 2.37) |  |
| Vascular dementia | PM_2.5-10_ |  |  |  |  |  |  |  |
|  |  | APOE |  |  |  |  |  |  |
|  |  | 0 | Ref | 1.02 (0.79 - 1.33) | 1.11 (0.86 - 1.43) | 1.18 (0.91 - 1.52) | 1.17 (0.91 - 1.51) | 0.325046556 |
|  |  | 1 | Ref | 1 (0.76 - 1.32) | 1.05 (0.8 - 1.38) | 0.94 (0.71 - 1.26) | 1.1 (0.84 - 1.45) |  |
|  |  | 2 | Ref | 1.52 (0.92 - 2.53) | 0.97 (0.54 - 1.72) | 0.87 (0.48 - 1.58) | 0.98 (0.55 - 1.76) |  |
| MCI | Air pollution score |  |  |  |  |  |  |  |
|  |  | APOE |  |  |  |  |  |  |
|  |  | 0 | Ref | 1.43 (1 - 2.06) | 1.35 (0.94 - 1.96) | 1.7 (1.19 - 2.42) | 2.09 (1.47 - 2.95) | 0.645400925 |
|  |  | 1 | Ref | 1.04 (0.66 - 1.63) | 1.08 (0.69 - 1.7) | 1.6 (1.05 - 2.44) | 1.85 (1.22 - 2.81) |  |
|  |  | 2 | Ref | 0.54 (0.2 - 1.43) | 0.95 (0.42 - 2.18) | 0.77 (0.31 - 1.9) | 1.41 (0.62 - 3.2) |  |
| MCI | NO_2_ |  |  |  |  |  |  |  |
|  |  | APOE |  |  |  |  |  |  |
|  |  | 0 | Ref | 1.45 (1 - 2.08) | 1.54 (1.07 - 2.21) | 1.7 (1.19 - 2.44) | 2.05 (1.44 - 2.92) | 0.263044989 |
|  |  | 1 | Ref | 1.13 (0.74 - 1.73) | 0.98 (0.63 - 1.52) | 1.29 (0.85 - 1.97) | 1.61 (1.06 - 2.45) |  |
|  |  | 2 | Ref | 0.47 (0.18 - 1.22) | 0.49 (0.19 - 1.29) | 1.11 (0.51 - 2.41) | 1.01 (0.44 - 2.33) |  |
| MCI | NO |  |  |  |  |  |  |  |
|  |  | APOE |  |  |  |  |  |  |
|  |  | 0 | Ref | 1.33 (0.93 - 1.92) | 1.22 (0.84 - 1.77) | 1.64 (1.15 - 2.33) | 2.09 (1.48 - 2.94) | 0.225081948 |
|  |  | 1 | Ref | 0.83 (0.54 - 1.28) | 0.77 (0.5 - 1.21) | 1.29 (0.86 - 1.92) | 1.48 (0.99 - 2.19) |  |
|  |  | 2 | Ref | 0.32 (0.11 - 0.98) | 0.9 (0.41 - 2) | 0.87 (0.39 - 1.96) | 0.95 (0.41 - 2.2) |  |
| MCI | PM_10_ |  |  |  |  |  |  |  |
|  |  | APOE |  |  |  |  |  |  |
|  |  | 0 | Ref | 1.43 (1.01 - 2.03) | 1.22 (0.85 - 1.75) | 1.49 (1.05 - 2.12) | 1.88 (1.35 - 2.63) | 0.185960683 |
|  |  | 1 | Ref | 0.79 (0.5 - 1.26) | 1.16 (0.76 - 1.78) | 1.55 (1.03 - 2.31) | 1.45 (0.97 - 2.18) |  |
|  |  | 2 | Ref | 0.65 (0.25 - 1.72) | 1.35 (0.59 - 3.1) | 0.71 (0.27 - 1.9) | 1.39 (0.6 - 3.23) |  |
| MCI | PM_2.5_ |  |  |  |  |  |  |  |
|  |  | APOE |  |  |  |  |  |  |
|  |  | 0 | Ref | 1.08 (0.75 - 1.56) | 1.38 (0.98 - 1.95) | 1.52 (1.08 - 2.13) | 1.74 (1.24 - 2.44) | 0.874986621 |
|  |  | 1 | Ref | 1.03 (0.66 - 1.62) | 1.09 (0.7 - 1.71) | 1.42 (0.93 - 2.17) | 1.87 (1.24 - 2.81) |  |
|  |  | 2 | Ref | 0.57 (0.22 - 1.46) | 0.93 (0.4 - 2.17) | 0.95 (0.4 - 2.22) | 1.18 (0.51 - 2.76) |  |
| MCI | PM_2.5-10_ |  |  |  |  |  |  |  |
|  |  | APOE |  |  |  |  |  |  |
|  |  | 0 | Ref | 0.84 (0.6 - 1.18) | 0.99 (0.71 - 1.36) | 1.2 (0.87 - 1.64) | 1.28 (0.94 - 1.75) | 0.7750814 |
|  |  | 1 | Ref | 0.85 (0.55 - 1.32) | 1.35 (0.91 - 2) | 1.18 (0.78 - 1.79) | 1.32 (0.88 - 1.96) |  |
|  |  | 2 | Ref | 1.55 (0.63 - 3.8) | 1.04 (0.39 - 2.79) | 1.34 (0.53 - 3.44) | 1.62 (0.65 - 4) |  |
|  | | | | | | | | |

**Supplementary Table S8. The analyses stratified by the healthy lifestyle according to the healthy lifestyle score**

| **Outcome** | **Expose** | **Subgroup** | **Q1** | **Q2** | **Q3** | **Q4** | **Q5** | **P for interaction** |
| --- | --- | --- | --- | --- | --- | --- | --- | --- |
| All-cause dementia | Air pollution score |  |  |  |  |  |  |  |
|  |  | HLS |  |  |  |  |  |  |
|  |  | 1 | Ref | 1.16 (1.04 - 1.28) | 1.27 (1.14 - 1.41) | 1.22 (1.09 - 1.35) | 1.34 (1.2 - 1.49) | 0.00085 |
|  |  | 2 | Ref | 0.91 (0.78 - 1.07) | 0.95 (0.81 - 1.12) | 1.17 (1 - 1.36) | 1.23 (1.05 - 1.44) |  |
|  |  | 3 | Ref | 1.04 (0.89 - 1.22) | 1 (0.85 - 1.18) | 1.07 (0.91 - 1.26) | 0.99 (0.84 - 1.17) |  |
| All-cause dementia | NO_2_ |  |  |  |  |  |  |  |
|  |  | HLS |  |  |  |  |  |  |
|  |  | 1 | Ref | 1.13 (1.01 - 1.25) | 1.23 (1.11 - 1.37) | 1.23 (1.11 - 1.36) | 1.3 (1.17 - 1.45) | 0.00358 |
|  |  | 2 | Ref | 0.91 (0.77 - 1.06) | 1.02 (0.87 - 1.19) | 1.08 (0.92 - 1.26) | 1.32 (1.13 - 1.54) |  |
|  |  | 3 | Ref | 1.01 (0.86 - 1.18) | 0.99 (0.84 - 1.16) | 1.03 (0.88 - 1.21) | 0.99 (0.84 - 1.17) |  |
| All-cause dementia | NO |  |  |  |  |  |  |  |
|  |  | HLS |  |  |  |  |  |  |
|  |  | 1 | Ref | 1.14 (1.03 - 1.27) | 1.13 (1.02 - 1.25) | 1.23 (1.11 - 1.36) | 1.25 (1.12 - 1.39) | 0.07309 |
|  |  | 2 | Ref | 0.98 (0.83 - 1.15) | 1.02 (0.87 - 1.19) | 1.16 (0.99 - 1.36) | 1.3 (1.11 - 1.52) |  |
|  |  | 3 | Ref | 1.04 (0.88 - 1.21) | 1.04 (0.89 - 1.22) | 1.11 (0.94 - 1.3) | 0.99 (0.84 - 1.18) |  |
| All-cause dementia | PM_10_ |  |  |  |  |  |  |  |
|  |  | HLS |  |  |  |  |  |  |
|  |  | 1 | Ref | 1.07 (0.97 - 1.19) | 1.21 (1.1 - 1.34) | 1.23 (1.11 - 1.37) | 1.15 (1.04 - 1.27) | 0.14931 |
|  |  | 2 | Ref | 1 (0.85 - 1.16) | 0.98 (0.84 - 1.15) | 1 (0.86 - 1.17) | 1.06 (0.91 - 1.24) |  |
|  |  | 3 | Ref | 1.07 (0.91 - 1.25) | 1.08 (0.92 - 1.27) | 1.06 (0.9 - 1.24) | 1 (0.84 - 1.18) |  |
| All-cause dementia | PM_2.5_ |  |  |  |  |  |  |  |
|  |  | HLS |  |  |  |  |  |  |
|  |  | 1 | Ref | 1.14 (1.02 - 1.26) | 1.17 (1.05 - 1.3) | 1.21 (1.09 - 1.34) | 1.25 (1.12 - 1.38) | 0.22995 |
|  |  | 2 | Ref | 1.03 (0.88 - 1.21) | 1.05 (0.9 - 1.24) | 1.22 (1.04 - 1.43) | 1.28 (1.1 - 1.5) |  |
|  |  | 3 | Ref | 1.07 (0.91 - 1.25) | 1.1 (0.94 - 1.29) | 1.15 (0.98 - 1.35) | 1.03 (0.87 - 1.22) |  |
| All-cause dementia | PM_2.5-10_ |  |  |  |  |  |  |  |
|  |  | HLS |  |  |  |  |  |  |
|  |  | 1 | Ref | 1.06 (0.96 - 1.17) | 1.09 (0.98 - 1.2) | 1.13 (1.02 - 1.25) | 1.09 (0.99 - 1.21) | 0.47435 |
|  |  | 2 | Ref | 1.03 (0.89 - 1.2) | 1 (0.86 - 1.17) | 0.93 (0.8 - 1.09) | 1.01 (0.87 - 1.18) |  |
|  |  | 3 | Ref | 1 (0.86 - 1.18) | 1 (0.85 - 1.17) | 1.07 (0.91 - 1.25) | 0.95 (0.81 - 1.12) |  |
| Alzheimer’s dementia | Air pollution score |  |  |  |  |  |  |  |
|  |  | HLS |  |  |  |  |  |  |
|  |  | 1 | Ref | 1.23 (1.04 - 1.46) | 1.29 (1.09 - 1.52) | 1.2 (1.01 - 1.43) | 1.37 (1.16 - 1.63) | 0.02512 |
|  |  | 2 | Ref | 0.99 (0.78 - 1.26) | 0.88 (0.68 - 1.12) | 1.21 (0.96 - 1.53) | 1.41 (1.11 - 1.77) |  |
|  |  | 3 | Ref | 0.95 (0.76 - 1.19) | 0.89 (0.7 - 1.12) | 0.98 (0.77 - 1.23) | 1.07 (0.85 - 1.36) |  |
| Alzheimer’s dementia | NO_2_ |  |  |  |  |  |  |  |
|  |  | HLS |  |  |  |  |  |  |
|  |  | 1 | Ref | 1.25 (1.06 - 1.48) | 1.3 (1.1 - 1.53) | 1.23 (1.04 - 1.46) | 1.31 (1.1 - 1.56) | 0.00214 |
|  |  | 2 | Ref | 0.92 (0.73 - 1.17) | 0.95 (0.75 - 1.21) | 0.99 (0.78 - 1.26) | 1.52 (1.21 - 1.91) |  |
|  |  | 3 | Ref | 0.84 (0.67 - 1.06) | 0.97 (0.77 - 1.21) | 0.87 (0.69 - 1.1) | 1.02 (0.81 - 1.29) |  |
| Alzheimer’s dementia | NO |  |  |  |  |  |  |  |
|  |  | HLS |  |  |  |  |  |  |
|  |  | 1 | Ref | 1.17 (0.98 - 1.38) | 1.19 (1.01 - 1.41) | 1.22 (1.03 - 1.44) | 1.29 (1.09 - 1.53) | 0.45087 |
|  |  | 2 | Ref | 1.01 (0.79 - 1.28) | 0.96 (0.75 - 1.22) | 1.3 (1.03 - 1.64) | 1.29 (1.02 - 1.64) |  |
|  |  | 3 | Ref | 0.99 (0.79 - 1.25) | 1 (0.8 - 1.27) | 1.08 (0.86 - 1.37) | 1.07 (0.84 - 1.36) |  |
| Alzheimer’s dementia | PM_10_ |  |  |  |  |  |  |  |
|  |  | HLS |  |  |  |  |  |  |
|  |  | 1 | Ref | 1.08 (0.92 - 1.28) | 1.25 (1.07 - 1.47) | 1.21 (1.03 - 1.43) | 1.15 (0.97 - 1.36) | 0.78968 |
|  |  | 2 | Ref | 1.05 (0.83 - 1.33) | 1.05 (0.83 - 1.34) | 1.06 (0.83 - 1.34) | 1.17 (0.93 - 1.48) |  |
|  |  | 3 | Ref | 1.05 (0.84 - 1.32) | 1.07 (0.85 - 1.35) | 1.01 (0.8 - 1.28) | 1.05 (0.83 - 1.33) |  |
| Alzheimer’s dementia | PM_2.5_ |  |  |  |  |  |  |  |
|  |  | HLS |  |  |  |  |  |  |
|  |  | 1 | Ref | 1.2 (1.02 - 1.42) | 1.19 (1.01 - 1.41) | 1.24 (1.05 - 1.46) | 1.28 (1.08 - 1.51) | 0.28277 |
|  |  | 2 | Ref | 0.97 (0.76 - 1.23) | 0.92 (0.72 - 1.18) | 1.17 (0.93 - 1.47) | 1.36 (1.08 - 1.72) |  |
|  |  | 3 | Ref | 1.05 (0.84 - 1.32) | 0.98 (0.78 - 1.24) | 1.04 (0.82 - 1.32) | 1.12 (0.88 - 1.41) |  |
| Alzheimer’s dementia | PM_2.5-10_ |  |  |  |  |  |  |  |
|  |  | HLS |  |  |  |  |  |  |
|  |  | 1 | Ref | 1.07 (0.91 - 1.26) | 1.1 (0.94 - 1.29) | 1.13 (0.96 - 1.32) | 1.06 (0.91 - 1.25) | 0.9689 |
|  |  | 2 | Ref | 1 (0.8 - 1.26) | 1 (0.79 - 1.26) | 0.96 (0.76 - 1.22) | 1.05 (0.83 - 1.31) |  |
|  |  | 3 | Ref | 0.95 (0.76 - 1.2) | 1.01 (0.81 - 1.27) | 1.01 (0.8 - 1.27) | 1.04 (0.83 - 1.31) |  |
| Vascular dementia | Air pollution score |  |  |  |  |  |  |  |
|  |  | HLS |  |  |  |  |  |  |
|  |  | 1 | Ref | 1.16 (0.93 - 1.44) | 1.34 (1.08 - 1.66) | 1.33 (1.07 - 1.66) | 1.37 (1.1 - 1.71) | 0.54955 |
|  |  | 2 | Ref | 0.93 (0.65 - 1.33) | 1.01 (0.71 - 1.44) | 1.2 (0.85 - 1.69) | 1.17 (0.82 - 1.66) |  |
|  |  | 3 | Ref | 1.19 (0.82 - 1.73) | 1.15 (0.78 - 1.68) | 1.06 (0.71 - 1.58) | 0.98 (0.65 - 1.49) |  |
| Vascular dementia | NO_2_ |  |  |  |  |  |  |  |
|  |  | HLS |  |  |  |  |  |  |
|  |  | 1 | Ref | 1.19 (0.96 - 1.49) | 1.41 (1.14 - 1.75) | 1.28 (1.03 - 1.59) | 1.38 (1.1 - 1.72) | 0.37618 |
|  |  | 2 | Ref | 0.88 (0.61 - 1.26) | 1.08 (0.76 - 1.52) | 1.15 (0.81 - 1.63) | 1.3 (0.91 - 1.84) |  |
|  |  | 3 | Ref | 1.13 (0.77 - 1.65) | 1.06 (0.72 - 1.56) | 1.24 (0.85 - 1.82) | 0.94 (0.62 - 1.43) |  |
| Vascular dementia | NO |  |  |  |  |  |  |  |
|  |  | HLS |  |  |  |  |  |  |
|  |  | 1 | Ref | 1.14 (0.91 - 1.42) | 1.33 (1.07 - 1.64) | 1.28 (1.03 - 1.58) | 1.32 (1.06 - 1.64) | 0.60725 |
|  |  | 2 | Ref | 1.14 (0.79 - 1.66) | 1.39 (0.97 - 1.99) | 1.27 (0.88 - 1.84) | 1.51 (1.05 - 2.17) |  |
|  |  | 3 | Ref | 0.86 (0.58 - 1.26) | 1.14 (0.79 - 1.63) | 0.89 (0.6 - 1.32) | 0.9 (0.6 - 1.34) |  |
| Vascular dementia | PM_10_ |  |  |  |  |  |  |  |
|  |  | HLS |  |  |  |  |  |  |
|  |  | 1 | Ref | 1.15 (0.93 - 1.42) | 1.19 (0.96 - 1.47) | 1.22 (0.99 - 1.51) | 1.32 (1.07 - 1.62) | 0.2799 |
|  |  | 2 | Ref | 1.21 (0.86 - 1.71) | 1.12 (0.79 - 1.59) | 0.87 (0.6 - 1.27) | 1.25 (0.89 - 1.77) |  |
|  |  | 3 | Ref | 1 (0.69 - 1.46) | 1.14 (0.79 - 1.65) | 0.88 (0.6 - 1.31) | 0.84 (0.56 - 1.26) |  |
| Vascular dementia | PM_2.5_ |  |  |  |  |  |  |  |
|  |  | HLS |  |  |  |  |  |  |
|  |  | 1 | Ref | 1.22 (0.98 - 1.52) | 1.2 (0.96 - 1.49) | 1.34 (1.08 - 1.66) | 1.32 (1.06 - 1.64) | 0.84181 |
|  |  | 2 | Ref | 1.33 (0.93 - 1.89) | 1.21 (0.84 - 1.73) | 1.26 (0.88 - 1.8) | 1.18 (0.82 - 1.71) |  |
|  |  | 3 | Ref | 1.14 (0.78 - 1.67) | 1.06 (0.72 - 1.56) | 1.2 (0.82 - 1.76) | 0.92 (0.6 - 1.39) |  |
| Vascular dementia | PM_2.5-10_ |  |  |  |  |  |  |  |
|  |  | HLS |  |  |  |  |  |  |
|  |  | 1 | Ref | 0.95 (0.77 - 1.17) | 1.02 (0.83 - 1.25) | 1.04 (0.85 - 1.28) | 1.16 (0.95 - 1.41) | 0.1119 |
|  |  | 2 | Ref | 0.99 (0.7 - 1.38) | 1.01 (0.72 - 1.42) | 0.86 (0.6 - 1.23) | 1.1 (0.79 - 1.53) |  |
|  |  | 3 | Ref | 1.64 (1.13 - 2.39) | 1.35 (0.91 - 2) | 1.09 (0.72 - 1.65) | 1.05 (0.69 - 1.59) |  |
| MCI | Air pollution score |  |  |  |  |  |  |  |
|  |  | HLS |  |  |  |  |  |  |
|  |  | 1 | Ref | 1.11 (0.81 - 1.54) | 1.27 (0.92 - 1.74) | 1.54 (1.13 - 2.08) | 1.8 (1.33 - 2.43) | 0.83171 |
|  |  | 2 | Ref | 0.95 (0.59 - 1.53) | 1.11 (0.7 - 1.77) | 1.15 (0.72 - 1.84) | 1.53 (0.98 - 2.39) |  |
|  |  | 3 | Ref | 0.66 (0.38 - 1.16) | 0.85 (0.5 - 1.43) | 1.23 (0.76 - 2.01) | 1.48 (0.92 - 2.38) |  |
| MCI | NO_2_ |  |  |  |  |  |  |  |
|  |  | HLS |  |  |  |  |  |  |
|  |  | 1 | Ref | 1.5 (1.1 - 2.06) | 1.17 (0.84 - 1.63) | 1.73 (1.27 - 2.36) | 1.84 (1.34 - 2.52) | 0.01462 |
|  |  | 2 | Ref | 0.98 (0.61 - 1.58) | 1.18 (0.75 - 1.86) | 0.97 (0.6 - 1.57) | 1.53 (0.98 - 2.4) |  |
|  |  | 3 | Ref | 0.43 (0.23 - 0.8) | 0.92 (0.56 - 1.51) | 1.01 (0.62 - 1.65) | 1.45 (0.92 - 2.29) |  |
| MCI | NO |  |  |  |  |  |  |  |
|  |  | HLS |  |  |  |  |  |  |
|  |  | 1 | Ref | 1.07 (0.78 - 1.48) | 0.96 (0.69 - 1.33) | 1.53 (1.14 - 2.06) | 1.77 (1.32 - 2.37) | 0.3011 |
|  |  | 2 | Ref | 1.05 (0.66 - 1.66) | 0.9 (0.56 - 1.47) | 1.18 (0.75 - 1.88) | 1.47 (0.94 - 2.29) |  |
|  |  | 3 | Ref | 0.69 (0.39 - 1.21) | 1.22 (0.75 - 2) | 0.93 (0.54 - 1.58) | 1.59 (0.99 - 2.55) |  |
| MCI | PM_10_ |  |  |  |  |  |  |  |
|  |  | HLS |  |  |  |  |  |  |
|  |  | 1 | Ref | 1.29 (0.95 - 1.76) | 1.3 (0.95 - 1.76) | 1.46 (1.08 - 1.98) | 1.63 (1.21 - 2.19) | 0.87938 |
|  |  | 2 | Ref | 0.87 (0.53 - 1.43) | 1.11 (0.69 - 1.78) | 1.39 (0.89 - 2.19) | 1.44 (0.92 - 2.26) |  |
|  |  | 3 | Ref | 1.23 (0.72 - 2.09) | 1.36 (0.8 - 2.3) | 1.17 (0.68 - 2.01) | 1.6 (0.96 - 2.67) |  |
| MCI | PM_2.5_ |  |  |  |  |  |  |  |
|  |  | HLS |  |  |  |  |  |  |
|  |  | 1 | Ref | 1 (0.72 - 1.39) | 1.41 (1.03 - 1.91) | 1.44 (1.07 - 1.95) | 1.7 (1.26 - 2.29) | 0.21876 |
|  |  | 2 | Ref | 0.96 (0.6 - 1.52) | 0.69 (0.41 - 1.15) | 1.37 (0.89 - 2.12) | 1.34 (0.87 - 2.09) |  |
|  |  | 3 | Ref | 1.1 (0.65 - 1.85) | 1.21 (0.72 - 2.02) | 1.01 (0.58 - 1.73) | 1.64 (1 - 2.7) |  |
| MCI | PM_2.5-10_ |  |  |  |  |  |  |  |
|  |  | HLS |  |  |  |  |  |  |
|  |  | 1 | Ref | 0.8 (0.59 - 1.08) | 1.2 (0.91 - 1.57) | 1.06 (0.8 - 1.41) | 1.2 (0.91 - 1.57) | 0.47084 |
|  |  | 2 | Ref | 0.98 (0.62 - 1.54) | 0.98 (0.62 - 1.55) | 1.23 (0.79 - 1.9) | 1.15 (0.74 - 1.78) |  |
|  |  | 3 | Ref | 1.23 (0.73 - 2.1) | 1.02 (0.59 - 1.78) | 1.69 (1.02 - 2.77) | 1.48 (0.88 - 2.47) |  |
| HLS: Healthy lifestyle score | | | | | | | | |

**Supplementary Table S9. The analyses stratified by other potential confounders**

| Outcome | Expose | Subgroup | Q1 | Q2 | Q3 | Q4 | Q5 | P for interaction |
| --- | --- | --- | --- | --- | --- | --- | --- | --- |
| All-cause dementia | Air pollution score |  |  |  |  |  |  |  |
|  |  | Sex |  |  |  |  |  |  |
|  |  | 0 | Ref | 1.04 (0.93 - 1.16) | 1.12 (1 - 1.25) | 1.16 (1.04 - 1.3) | 1.24 (1.1 - 1.38) | 0.803758274 |
|  |  | 1 | Ref | 1.09 (0.98 - 1.22) | 1.14 (1.03 - 1.27) | 1.16 (1.04 - 1.29) | 1.19 (1.07 - 1.33) |  |
|  |  | Age |  |  |  |  |  |  |
|  |  | 0 | Ref | 1.03 (0.83 - 1.28) | 0.97 (0.78 - 1.2) | 1.11 (0.91 - 1.36) | 1.06 (0.86 - 1.3) | 0.561933469 |
|  |  | 1 | Ref | 1.08 (0.99 - 1.17) | 1.16 (1.07 - 1.26) | 1.17 (1.08 - 1.27) | 1.25 (1.14 - 1.35) |  |
|  |  | Ethnicity |  |  |  |  |  |  |
|  |  | 0 | Ref | 1.75 (0.87 - 3.52) | 1.96 (1 - 3.82) | 1.88 (0.98 - 3.63) | 1.89 (0.99 - 3.61) | 0.550173501 |
|  |  | 1 | Ref | 1.06 (0.98 - 1.15) | 1.12 (1.04 - 1.21) | 1.15 (1.07 - 1.25) | 1.21 (1.12 - 1.31) |  |
|  |  | Education |  |  |  |  |  |  |
|  |  | 0 | Ref | 1.08 (0.96 - 1.21) | 1.22 (1.09 - 1.36) | 1.22 (1.09 - 1.37) | 1.26 (1.12 - 1.41) | 0.264458145 |
|  |  | 1 | Ref | 1.07 (0.96 - 1.18) | 1.05 (0.95 - 1.17) | 1.11 (1 - 1.23) | 1.18 (1.06 - 1.31) |  |
|  |  | CVD |  |  |  |  |  |  |
|  |  | 0 | Ref | 1.06 (0.97 - 1.15) | 1.1 (1.01 - 1.2) | 1.16 (1.06 - 1.26) | 1.21 (1.11 - 1.32) | 0.684529931 |
|  |  | 1 | Ref | 1.11 (0.93 - 1.34) | 1.24 (1.04 - 1.48) | 1.16 (0.96 - 1.38) | 1.21 (1.01 - 1.45) |  |
|  |  | Hypertension |  |  |  |  |  |  |
|  |  | 0 | Ref | 1.07 (0.97 - 1.18) | 1.16 (1.05 - 1.28) | 1.19 (1.08 - 1.31) | 1.17 (1.06 - 1.3) | 0.170665688 |
|  |  | 1 | Ref | 1.07 (0.95 - 1.21) | 1.09 (0.97 - 1.23) | 1.13 (1 - 1.28) | 1.27 (1.12 - 1.43) |  |
|  |  | Diabetes |  |  |  |  |  |  |
|  |  | 0 | Ref | 1.08 (0.99 - 1.17) | 1.12 (1.03 - 1.21) | 1.16 (1.07 - 1.26) | 1.22 (1.12 - 1.32) | 0.779740625 |
|  |  | 1 | Ref | 1.01 (0.8 - 1.27) | 1.21 (0.97 - 1.51) | 1.17 (0.94 - 1.45) | 1.21 (0.98 - 1.51) |  |
| Alzheimer’s dementia | Air pollution score |  |  |  |  |  |  |  |
|  |  | Sex |  |  |  |  |  |  |
|  |  | 0 | Ref | 1.08 (0.92 - 1.28) | 1.08 (0.91 - 1.28) | 1.23 (1.04 - 1.45) | 1.38 (1.17 - 1.63) | 0.333292718 |
|  |  | 1 | Ref | 1.09 (0.92 - 1.28) | 1.04 (0.88 - 1.23) | 1.03 (0.86 - 1.22) | 1.17 (0.99 - 1.39) |  |
|  |  | Age |  |  |  |  |  |  |
|  |  | 0 | Ref | 0.84 (0.58 - 1.2) | 0.68 (0.46 - 0.99) | 0.95 (0.67 - 1.34) | 0.96 (0.68 - 1.34) | 0.175444807 |
|  |  | 1 | Ref | 1.12 (0.99 - 1.27) | 1.12 (0.99 - 1.26) | 1.15 (1.01 - 1.3) | 1.32 (1.17 - 1.5) |  |
|  |  | Ethnicity |  |  |  |  |  |  |
|  |  | 0 | Ref | 2.38 (0.69 - 8.23) | 2.33 (0.7 - 7.82) | 2.6 (0.8 - 8.5) | 2.39 (0.74 - 7.71) | 0.605303113 |
|  |  | 1 | Ref | 1.08 (0.96 - 1.21) | 1.05 (0.93 - 1.19) | 1.11 (0.98 - 1.25) | 1.28 (1.13 - 1.44) |  |
|  |  | Education |  |  |  |  |  |  |
|  |  | 0 | Ref | 1.12 (0.94 - 1.33) | 1.14 (0.96 - 1.36) | 1.25 (1.06 - 1.48) | 1.35 (1.14 - 1.61) | 0.45907649 |
|  |  | 1 | Ref | 1.07 (0.91 - 1.25) | 1 (0.84 - 1.18) | 1.01 (0.85 - 1.19) | 1.22 (1.03 - 1.44) |  |
|  |  | CVD |  |  |  |  |  |  |
|  |  | 0 | Ref | 1.04 (0.92 - 1.18) | 0.98 (0.86 - 1.12) | 1.1 (0.97 - 1.25) | 1.27 (1.12 - 1.45) | 0.013645224 |
|  |  | 1 | Ref | 1.36 (1 - 1.85) | 1.55 (1.15 - 2.1) | 1.28 (0.93 - 1.75) | 1.3 (0.95 - 1.79) |  |
|  |  | Hypertension |  |  |  |  |  |  |
|  |  | 0 | Ref | 1.07 (0.93 - 1.24) | 1.06 (0.91 - 1.23) | 1.16 (1 - 1.35) | 1.2 (1.03 - 1.4) | 0.267314578 |
|  |  | 1 | Ref | 1.11 (0.92 - 1.35) | 1.07 (0.88 - 1.3) | 1.07 (0.88 - 1.31) | 1.38 (1.14 - 1.67) |  |
|  |  | Diabetes |  |  |  |  |  |  |
|  |  | 0 | Ref | 1.08 (0.95 - 1.22) | 1.02 (0.9 - 1.16) | 1.13 (0.99 - 1.28) | 1.28 (1.13 - 1.45) | 0.235794561 |
|  |  | 1 | Ref | 1.16 (0.8 - 1.68) | 1.4 (0.98 - 2) | 1.12 (0.78 - 1.62) | 1.26 (0.88 - 1.82) |  |
| Vascular dementia | Air pollution score |  |  |  |  |  |  |  |
|  |  | Sex |  |  |  |  |  |  |
|  |  | 0 | Ref | 1.24 (0.95 - 1.62) | 1.2 (0.92 - 1.57) | 1.27 (0.97 - 1.65) | 1.44 (1.11 - 1.87) | 0.764330203 |
|  |  | 1 | Ref | 1.14 (0.92 - 1.41) | 1.26 (1.01 - 1.55) | 1.22 (0.98 - 1.51) | 1.24 (1 - 1.55) |  |
|  |  | Age |  |  |  |  |  |  |
|  |  | 0 | Ref | 1.72 (0.97 - 3.03) | 1.06 (0.57 - 1.96) | 1.65 (0.95 - 2.89) | 1.5 (0.86 - 2.63) | 0.211216879 |
|  |  | 1 | Ref | 1.14 (0.95 - 1.36) | 1.26 (1.06 - 1.49) | 1.2 (1.01 - 1.43) | 1.31 (1.09 - 1.56) |  |
|  |  | Ethnicity |  |  |  |  |  |  |
|  |  | 0 | Ref | 1.59 (0.33 - 7.65) | 2.13 (0.48 - 9.38) | 1.49 (0.34 - 6.47) | 1.74 (0.41 - 7.28) | 0.880426561 |
|  |  | 1 | Ref | 1.18 (0.99 - 1.39) | 1.22 (1.03 - 1.44) | 1.24 (1.05 - 1.47) | 1.32 (1.11 - 1.56) |  |
|  |  | Education |  |  |  |  |  |  |
|  |  | 0 | Ref | 1.28 (1 - 1.65) | 1.55 (1.22 - 1.98) | 1.36 (1.06 - 1.75) | 1.51 (1.18 - 1.93) | 0.064636492 |
|  |  | 1 | Ref | 1.12 (0.89 - 1.4) | 0.97 (0.76 - 1.23) | 1.16 (0.92 - 1.47) | 1.18 (0.93 - 1.49) |  |
|  |  | CVD |  |  |  |  |  |  |
|  |  | 0 | Ref | 1.27 (1.04 - 1.55) | 1.26 (1.04 - 1.54) | 1.25 (1.02 - 1.52) | 1.38 (1.13 - 1.69) | 0.578892357 |
|  |  | 1 | Ref | 0.96 (0.7 - 1.32) | 1.13 (0.83 - 1.53) | 1.16 (0.86 - 1.58) | 1.14 (0.84 - 1.54) |  |
|  |  | Hypertension |  |  |  |  |  |  |
|  |  | 0 | Ref | 1.34 (1.05 - 1.71) | 1.37 (1.07 - 1.75) | 1.5 (1.18 - 1.92) | 1.53 (1.19 - 1.96) | 0.441608461 |
|  |  | 1 | Ref | 1.05 (0.84 - 1.33) | 1.12 (0.9 - 1.41) | 1.04 (0.83 - 1.31) | 1.16 (0.92 - 1.46) |  |
|  |  | Diabetes |  |  |  |  |  |  |
|  |  | 0 | Ref | 1.17 (0.97 - 1.41) | 1.18 (0.98 - 1.42) | 1.3 (1.08 - 1.57) | 1.35 (1.12 - 1.63) | 0.22128681 |
|  |  | 1 | Ref | 1.2 (0.82 - 1.76) | 1.42 (0.98 - 2.05) | 1.02 (0.69 - 1.51) | 1.23 (0.84 - 1.79) |  |
| MCI | Air pollution score |  |  |  |  |  |  |  |
|  |  | Sex |  |  |  |  |  |  |
|  |  | 0 | Ref | 0.96 (0.66 - 1.4) | 1.13 (0.79 - 1.62) | 1.36 (0.96 - 1.92) | 1.86 (1.34 - 2.6) | 0.389122721 |
|  |  | 1 | Ref | 1.16 (0.85 - 1.59) | 1.13 (0.83 - 1.55) | 1.5 (1.11 - 2.03) | 1.56 (1.15 - 2.11) |  |
|  |  | Age |  |  |  |  |  |  |
|  |  | 0 | Ref | 0.76 (0.41 - 1.39) | 1.39 (0.83 - 2.34) | 2.07 (1.28 - 3.36) | 1.7 (1.04 - 2.78) | 0.015742124 |
|  |  | 1 | Ref | 1.16 (0.89 - 1.5) | 1.08 (0.83 - 1.41) | 1.27 (0.98 - 1.65) | 1.72 (1.34 - 2.21) |  |
|  |  | Ethnicity |  |  |  |  |  |  |
|  |  | 0 | Ref | 1.25 (0.24 - 6.45) | 1.76 (0.39 - 7.96) | 1.39 (0.31 - 6.15) | 1.57 (0.37 - 6.66) | 0.676624189 |
|  |  | 1 | Ref | 1.07 (0.84 - 1.36) | 1.11 (0.87 - 1.41) | 1.46 (1.15 - 1.83) | 1.73 (1.38 - 2.17) |  |
|  |  | Education |  |  |  |  |  |  |
|  |  | 0 | Ref | 1.06 (0.72 - 1.57) | 1.16 (0.8 - 1.69) | 1.62 (1.14 - 2.32) | 1.8 (1.27 - 2.57) | 0.807207385 |
|  |  | 1 | Ref | 1.09 (0.8 - 1.48) | 1.12 (0.83 - 1.53) | 1.3 (0.96 - 1.75) | 1.62 (1.21 - 2.16) |  |
|  |  | CVD |  |  |  |  |  |  |
|  |  | 0 | Ref | 0.98 (0.76 - 1.28) | 1.04 (0.8 - 1.35) | 1.35 (1.05 - 1.73) | 1.65 (1.3 - 2.11) | 0.408179086 |
|  |  | 1 | Ref | 1.61 (0.89 - 2.91) | 1.73 (0.97 - 3.11) | 1.99 (1.12 - 3.54) | 2 (1.13 - 3.56) |  |
|  |  | Hypertension |  |  |  |  |  |  |
|  |  | 0 | Ref | 0.79 (0.58 - 1.08) | 0.84 (0.61 - 1.14) | 1.27 (0.95 - 1.68) | 1.44 (1.09 - 1.9) | 0.019610794 |
|  |  | 1 | Ref | 1.7 (1.15 - 2.52) | 1.79 (1.22 - 2.65) | 1.88 (1.27 - 2.77) | 2.32 (1.59 - 3.39) |  |
|  |  | Diabetes |  |  |  |  |  |  |
|  |  | 0 | Ref | 1.03 (0.79 - 1.33) | 1.1 (0.86 - 1.42) | 1.4 (1.1 - 1.79) | 1.65 (1.3 - 2.09) | 0.929916812 |
|  |  | 1 | Ref | 1.47 (0.73 - 2.97) | 1.41 (0.7 - 2.86) | 1.8 (0.91 - 3.53) | 2.18 (1.13 - 4.21) |  |

**Supplementary Table S10. The sensitivity analysis by excluding the cases diagnosed in the first two years of follow-up, adjusted by potential confounders**

|  | Q1 (39.22, 49.82) | Q2 (49.82, 54.61) | Q3 (49.82, 54.61) | Q4 (58.38, 63.09) | Q5 (63.09, 157.77) | HR, std | P for trend |
| --- | --- | --- | --- | --- | --- | --- | --- |
| **All-cause dementia** |  |  |  |  |  |  |  |
| Case/person-years | 1210/1153126 | 1363/1149024 | 1440/1143958 | 1434/1142229 | 1431/1147737 |  |  |
| Model1 | Reference | 1.15 (1.06 - 1.24) | 1.26 (1.16 - 1.36) | 1.33 (1.24 - 1.44) | 1.44 (1.34 - 1.56) | 1.13 (1.1 - 1.15) | 2.28496E-24 |
| Model2 | Reference | 1.08 (1 - 1.17) | 1.15 (1.06 - 1.24) | 1.19 (1.1 - 1.29) | 1.26 (1.16 - 1.36) | 1.08 (1.05 - 1.1) | 7.10015E-10 |
| Model3 | Reference | 1.07 (0.99 - 1.15) | 1.13 (1.05 - 1.22) | 1.16 (1.07 - 1.25) | 1.21 (1.12 - 1.31) | 1.06 (1.04 - 1.09) | 2.41363E-07 |
| **Alzheimer’s dementia** |  |  |  |  |  |  |  |
| Case/person-years | 523/1154638 | 593/1150382 | 567/1146023 | 582/1144406 | 622/1149902 |  |  |
| Model1 | Reference | 1.15 (1.02 - 1.3) | 1.14 (1.01 - 1.29) | 1.25 (1.11 - 1.41) | 1.45 (1.29 - 1.63) | 1.12 (1.09 - 1.16) | 3.29767E-10 |
| Model2 | Reference | 1.09 (0.97 - 1.23) | 1.05 (0.94 - 1.19) | 1.14 (1.01 - 1.28) | 1.3 (1.16 - 1.47) | 1.09 (1.05 - 1.13) | 3.08039E-05 |
| Model3 | Reference | 1.09 (0.97 - 1.23) | 1.05 (0.93 - 1.19) | 1.13 (1 - 1.27) | 1.28 (1.14 - 1.45) | 1.08 (1.04 - 1.12) | 9.04208E-05 |
| **Vascular dementia** |  |  |  |  |  |  |  |
| Case/person-years | 243/1155235 | 309/1151665 | 320/1147325 | 316/1145299 | 325/1149475 |  |  |
| Model1 | Reference | 1.28 (1.09 - 1.52) | 1.4 (1.18 - 1.65) | 1.46 (1.24 - 1.73) | 1.62 (1.37 - 1.92) | 1.15 (1.1 - 1.21) | 5.88679E-09 |
| Model2 | Reference | 1.2 (1.01 - 1.42) | 1.24 (1.05 - 1.47) | 1.27 (1.07 - 1.5) | 1.36 (1.15 - 1.61) | 1.09 (1.04 - 1.15) | 0.00060957 |
| Model3 | Reference | 1.17 (0.99 - 1.38) | 1.2 (1.01 - 1.42) | 1.2 (1.01 - 1.42) | 1.25 (1.06 - 1.49) | 1.06 (1.01 - 1.12) | 0.015667213 |
| **MCI** |  |  |  |  |  |  |  |
| Case/person-years | 125/1155244 | 142/1151421 | 153/1147317 | 189/1145741 | 229/1149915 |  |  |
| Model1 | Reference | 1.15 (0.91 - 1.47) | 1.29 (1.02 - 1.63) | 1.67 (1.33 - 2.1) | 2.14 (1.72 - 2.66) | 1.26 (1.19 - 1.34) | 6.72599E-15 |
| Model2 | Reference | 1.09 (0.86 - 1.39) | 1.17 (0.92 - 1.48) | 1.48 (1.18 - 1.86) | 1.8 (1.44 - 2.25) | 1.21 (1.13 - 1.28) | 2.05487E-09 |
| Model3 | Reference | 1.07 (0.84 - 1.36) | 1.14 (0.9 - 1.45) | 1.43 (1.14 - 1.8) | 1.71 (1.37 - 2.14) | 1.19 (1.12 - 1.27) | 2.88334E-08 |
| Model1 was adjusted for age and sex.  Model2 was adjusted for age, sex, ethnicity, income, employment, education, drink, and smoke.  Model3 was adjusted for age, sex, ethnicity, income, employment, education, drink, smoke, MET, BMI, SBP, hypertension, CVD, and diabetes. | | | | | | | |

**Supplementary Table S11. The sensitivity analysis by restricting the follow-up duration to at least five years, adjusted by potential confounders**

|  | Q1 (39.22, 49.82) | Q2 (49.82, 54.61) | Q3 (49.82, 54.61) | Q4 (58.38, 63.09) | Q5 (63.09, 157.77) | HR, std | P for trend |
| --- | --- | --- | --- | --- | --- | --- | --- |
| **All-cause dementia** |  |  |  |  |  |  |  |
| Case/person-years | 1025/925475 | 1131/921835 | 1195/918067 | 1233/916042 | 1238/919426 |  |  |
| Model1 | Reference | 1.13 (1.04 - 1.23) | 1.22 (1.13 - 1.33) | 1.33 (1.23 - 1.45) | 1.43 (1.31 - 1.55) | 1.12 (1.1 - 1.15) | 8.57531E-21 |
| Model2 | Reference | 1.07 (0.98 - 1.16) | 1.13 (1.04 - 1.23) | 1.2 (1.11 - 1.31) | 1.26 (1.16 - 1.37) | 1.08 (1.05 - 1.11) | 1.57059E-09 |
| Model3 | Reference | 1.06 (0.97 - 1.15) | 1.11 (1.02 - 1.21) | 1.18 (1.08 - 1.28) | 1.22 (1.12 - 1.32) | 1.07 (1.04 - 1.1) | 1.95249E-07 |
| **Alzheimer’s dementia** |  |  |  |  |  |  |  |
| Case/person-years | 447/926682 | 496/923177 | 470/919770 | 504/918016 | 545/921306 |  |  |
| Model1 | Reference | 1.13 (1 - 1.29) | 1.1 (0.97 - 1.25) | 1.25 (1.1 - 1.42) | 1.44 (1.27 - 1.63) | 1.12 (1.08 - 1.16) | 5.94728E-09 |
| Model2 | Reference | 1.08 (0.95 - 1.22) | 1.02 (0.9 - 1.17) | 1.14 (1 - 1.3) | 1.3 (1.14 - 1.47) | 1.08 (1.04 - 1.13) | 7.04346E-05 |
| Model3 | Reference | 1.08 (0.95 - 1.22) | 1.02 (0.9 - 1.17) | 1.14 (1 - 1.29) | 1.28 (1.13 - 1.46) | 1.08 (1.04 - 1.12) | 0.000151662 |
| **Vascular dementia** |  |  |  |  |  |  |  |
| Case/person-years | 211/927036 | 256/924102 | 270/920708 | 264/918776 | 272/921312 |  |  |
| Model1 | Reference | 1.23 (1.02 - 1.47) | 1.35 (1.13 - 1.62) | 1.38 (1.15 - 1.66) | 1.51 (1.26 - 1.81) | 1.13 (1.08 - 1.2) | 3.7428E-06 |
| Model2 | Reference | 1.15 (0.96 - 1.39) | 1.22 (1.02 - 1.46) | 1.23 (1.02 - 1.47) | 1.31 (1.09 - 1.57) | 1.08 (1.03 - 1.15) | 0.00465116 |
| Model3 | Reference | 1.12 (0.94 - 1.35) | 1.18 (0.98 - 1.41) | 1.16 (0.97 - 1.4) | 1.21 (1.01 - 1.45) | 1.06 (1 - 1.12) | 0.051980966 |
| **MCI** |  |  |  |  |  |  |  |
| Case/person-years | 111/927141 | 120/924018 | 117/920521 | 147/919341 | 197/921640 |  |  |
| Model1 | Reference | 1.1 (0.85 - 1.42) | 1.1 (0.85 - 1.43) | 1.44 (1.13 - 1.84) | 2.02 (1.6 - 2.55) | 1.24 (1.17 - 1.33) | 6.34955E-11 |
| Model2 | Reference | 1.04 (0.8 - 1.35) | 1.01 (0.78 - 1.31) | 1.28 (1 - 1.64) | 1.71 (1.35 - 2.17) | 1.18 (1.11 - 1.27) | 7.77065E-07 |
| Model3 | Reference | 1.02 (0.79 - 1.32) | 0.99 (0.76 - 1.28) | 1.25 (0.97 - 1.6) | 1.63 (1.29 - 2.07) | 1.17 (1.09 - 1.25) | 4.79734E-06 |
| Model1 was adjusted for age and sex.  Model2 was adjusted for age, sex, ethnicity, income, employment, education, drink, and smoke.  Model3 was adjusted for age, sex, ethnicity, income, employment, education, drink, smoke, MET, BMI, SBP, hypertension, CVD, and diabetes. | | | | | | | |

**Supplementary Table S12. The sensitivity analysis by excluding the influence from other mixed confounders**

|  | Q1 (39.22, 49.82) | Q2 (49.82, 54.61) | Q3 (49.82, 54.61) | Q4 (58.38, 63.09) | Q5 (63.09, 157.77) | HR, std | P for trend |
| --- | --- | --- | --- | --- | --- | --- | --- |
| **All-cause dementia** |  |  |  |  |  |  |  |
| Case/person-years | 1025/925475 | 1131/921835 | 1195/918067 | 1233/916042 | 1238/919426 |  |  |
| Model1 | Reference | 1.06 (0.97 - 1.15) | 1.12 (1.03 - 1.22) | 1.19 (1.09 - 1.29) | 1.23 (1.13 - 1.34) | 1.07 (1.05 - 1.1) | 3.09483E-08 |
| Model2 | Reference | 1.05 (0.97 - 1.14) | 1.11 (1.02 - 1.21) | 1.18 (1.08 - 1.28) | 1.21 (1.11 - 1.32) | 1.07 (1.04 - 1.09) | 2.82759E-07 |
| Model3 | Reference | 1.07 (0.98 - 1.16) | 1.15 (1.06 - 1.26) | 1.22 (1.12 - 1.33) | 1.23 (1.13 - 1.35) | 1.07 (1.04 - 1.1) | 1.55924E-07 |
| **Alzheimer’s dementia** |  |  |  |  |  |  |  |
| Case/person-years | 447/926682 | 496/923177 | 470/919770 | 504/918016 | 545/921306 |  |  |
| Model1 | Reference | 1.08 (0.95 - 1.22) | 1.03 (0.9 - 1.17) | 1.14 (1 - 1.3) | 1.29 (1.14 - 1.47) | 1.08 (1.04 - 1.13) | 8.49529E-05 |
| Model2 | Reference | 1.07 (0.94 - 1.22) | 1.02 (0.9 - 1.16) | 1.13 (1 - 1.29) | 1.28 (1.13 - 1.45) | 1.08 (1.04 - 1.12) | 0.000172089 |
| Model3 | Reference | 1.09 (0.96 - 1.24) | 1.06 (0.93 - 1.21) | 1.17 (1.02 - 1.34) | 1.27 (1.11 - 1.46) | 1.07 (1.03 - 1.12) | 0.000684371 |
| **Vascular dementia** |  |  |  |  |  |  |  |
| Case/person-years | 211/927036 | 256/924102 | 270/920708 | 264/918776 | 272/921312 |  |  |
| Model1 | Reference | 1.13 (0.94 - 1.36) | 1.2 (1 - 1.43) | 1.18 (0.99 - 1.42) | 1.25 (1.04 - 1.5) | 1.07 (1.01 - 1.13) | 0.023169305 |
| Model2 | Reference | 1.12 (0.93 - 1.34) | 1.17 (0.98 - 1.4) | 1.16 (0.97 - 1.39) | 1.2 (1 - 1.44) | 1.05 (1 - 1.11) | 0.061426757 |
| Model3 | Reference | 1.11 (0.92 - 1.33) | 1.17 (0.97 - 1.4) | 1.15 (0.96 - 1.39) | 1.23 (1.01 - 1.49) | 1.06 (1 - 1.13) | 0.048660206 |
| **MCI** |  |  |  |  |  |  |  |
| Case/person-years | 111/927141 | 120/924018 | 117/920521 | 147/919341 | 197/921640 |  |  |
| Model1 | Reference | 1.03 (0.8 - 1.34) | 1 (0.77 - 1.29) | 1.26 (0.98 - 1.61) | 1.66 (1.31 - 2.11) | 1.17 (1.1 - 1.26) | 2.38929E-06 |
| Model2 | Reference | 1.02 (0.79 - 1.32) | 0.99 (0.76 - 1.28) | 1.25 (0.97 - 1.6) | 1.63 (1.28 - 2.07) | 1.17 (1.09 - 1.25) | 4.56204E-06 |
| Model3 | Reference | 1.07 (0.82 - 1.39) | 1.04 (0.79 - 1.35) | 1.24 (0.96 - 1.61) | 1.36 (1.05 - 1.76) | 1.07 (0.98 - 1.15) | 0.010460073 |
| Model1 was adjusted for age and sex.  Model2 was adjusted for age, sex, ethnicity, income, employment, education, drink, and smoke.  Model3 was adjusted for age, sex, ethnicity, income, employment, education, drink, smoke, MET, BMI, SBP, hypertension, CVD, and diabetes. | | | | | | | |
